# Effect of the polysaccharide capsule and its heptose on the resistance of *Campylobacter jejuni* to innate immune defenses

**DOI:** 10.1002/mbo3.1400

**Published:** 2024-02-20

**Authors:** Matthew Myles, Heba Barnawi, Mahmoud Mahmoudpour, Sargon Shlimon, Adrienne Chang, Daniel Zimmermann, Chiwon Choi, Najwa Zebian, Carole Creuzenet

**Affiliations:** ^1^ Microbiology and Immunology The University of Western Ontario London Ontario Canada

**Keywords:** *Campylobacter jejuni*, capsule, heptose, innate immune defenses, macrophages, redox stress

## Abstract

*Campylobacter jejuni* is a commensal in many animals but causes diarrhea in humans. Its polysaccharide capsule contributes to host colonization and virulence in a strain‐ and model‐specific manner. We investigated if the capsule and its heptose are important for interactions of strain NCTC 11168 with various hosts and their innate immune defenses. We determined that they support bacterial survival in *Drosophila melanogaster* and enhance virulence in *Galleria mellonella*. We showed that the capsule had limited antiphagocytic activity in human and chicken macrophages, decreased adherence to chicken macrophages, and decreased intracellular survival in both macrophages. In contrast, the heptose increased uptake by chicken macrophages and supported adherence to human macrophages and survival within them. While the capsule triggered nitric oxide production in chicken macrophages, the heptose mitigated this and protected against nitrosative assault. Finally, the *C. jejuni* strain NCTC 11168 elicited strong cytokine production in both macrophages but quenched ROS production independently from capsule and heptose, and while the capsule and heptose did not protect against oxidative assault, they favored growth in biofilms under oxidative stress. This study shows that the wild‐type capsule with its heptose is optimized to resist innate defenses in strain NCTC 11168 often via antagonistic effects of the capsule and its heptose.

## Graphical abstract

This multispecies study shows that *Campylobacter jejuni*'s capsule and its heptose are double edge swords that can protect *C. jejuni* against some host defenses while they enhance the activation of others. The fine‐tuning of capsule composition may allow better survival at different stages of colonization in commensal versus pathogenic models.

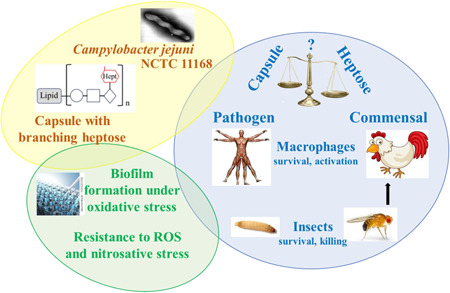

## 1 INTRODUCTION

### 1.1 *Campylobacter jejuni* as a human pathogen

*C. jejuni* is a gram‐negative, microaerophilic bacterium that can cause a food‐ or water‐borne enteritis called campylobacteriosis in humans (Silva et al., 2011). It causes 60%–80% of all pathogenic *Campylobacter* infections in developed countries (Kaakoush et al., 2015). Infections clear spontaneously in most cases, though increased risk comes with the rise of antibiotic resistance that led the World Health Organization to list Campylobacters as high‐priority pathogens (Control, 2019; Hakanen, 2003; Nunes et al., 2023; Tacconelli et al., 2018). *C. jejuni* is naturally found as a commensal in poultry, wild birds, and cattle. Human infections can be acquired by consuming unpasteurized milk, contaminated water or insufficiently cooked contaminated meat (Brown et al., 1988; Robinson & Jones, 1981). In Canada, consumption of contaminated undercooked chicken meat contributes to ~65% *Campylobacter* infections (Agunos et al., 2014; Skarp et al., 2016) and *C*. *jejuni* can be isolated from over 32% of broiler chicken meat samples tested (Agunos et al., 2014).

### 1.2 Role of capsule in virulence of *C. jejuni*

*C. jejuni* uses a wide range of virulence factors to survive in the environment and to colonize various hosts in which it can develop commensal or pathogenic relationships (Burnham & Hendrixson, 2018; Christensen et al., 2009; Konkel et al., 2004; Szymanski et al., 2002). This includes the capsule polysaccharide that surrounds the bacterium and is the focus of this study. Several groups are targeting the capsule for vaccine development (Guerry et al., 2012; Maue et al., 2014; Ramakrishnan et al., 2019) as clinical isolates express elevated levels of capsular gene transcripts compared to laboratory strains (Kovacs et al., 2020) and the capsule increases host epithelial cells invasion and gut colonization, and decreases sensitivity to killing by serum or antimicrobial peptides (Bachtiar et al., 2007; Bacon et al., 2001; Guerry et al., 2012; Jones et al., 2004; Keo et al., 2011; Maue et al., 2013, 2014; Novik et al., 2010; Wong et al., 2015). However, its importance seems strain‐ and model‐specific and does not follow the paradigm established in other bacteria (Willis & Whitfield, 2013). Indeed, in *C. jejuni* strain 81–176, an a‐capsular *kpsM* mutant showed impaired colonization in ferrets and decreased invasion of intestinal cells (Bacon et al., 2001; Maue et al., 2013, 2014) while in strain NCTC 11186, the *kpsM* mutant only reduced intestinal colonization of chicken slightly and was more adhesive and invasive to epithelial cells (Jones et al., 2004; Wong et al., 2015). Furthermore, the capsule does not have the expected strong antiphagocytic role as it still allows the uptake by macrophages (Day et al., 2000; Hickey et al., 2005; Kiehlbauch et al., 1985; Myszewski & Stern, 1991; Wassenaar et al., 1997; Watson & Galán, 2008; Wong et al., 2015). Also, the capsule does not prevent signaling from underlying structures such as the lipooligosaccharide, as a strong pro‐inflammatory response to *C. jejuni* occurs (Stephenson et al., 2013) and the capsule only partly mitigates the immune response in several strains and cell types (Maue et al., 2013; Rose et al., 2012).

### 1.3 Unique presence of modified heptoses in the *C. jejuni* capsule

**Figure 1:**
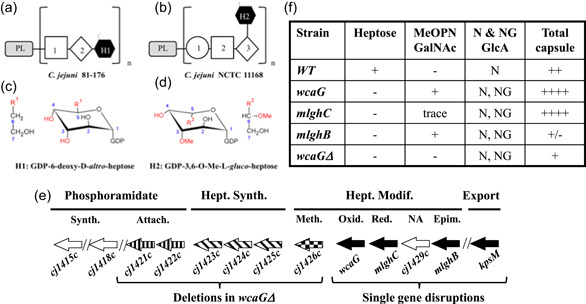
Summary of capsule composition and heptose content of two  *Campylobacter jejuni* isolates and the series of mutants used in this study (a, b). Schematic structure of the capsule in *C. jejuni* 81–176 and NCTC 11168 respectively, with their phospholipid anchor (PL). In strain 81–176, the repeating unit is made of D‐GlcNAc (1), D‐Gal (2) and modified 6‐deoxy‐*altro*‐heptose (H1). In strain NCTC 11168, the repeating unit is made of D‐rib*f* (1), D‐Gal*f*NAc (2), and D‐Glc*p*A (3), with a side branch of modified 3,6‐OMe‐L‐*gluco*‐heptose (H2). (c and d) Chemical structure of the specific modified heptose present in each strain. The data for panels a–d are based on Aspinall et al. (1992), Corcoran et al. (2006), St Michael et al. (2002). (e) Schematic view of the capsular gene cluster organization and main associated functions relevant to this study. Each mutant is made by single gene disruption (black bars) but the *wacGΔ* mutant also includes a large deletion from genes *cj1421c* to *cj1426c* (stripes, hatching, and checkboard patterns). Genes unaffected in all mutants are shown as white bars. Drawing not to scale. Hept., heptose; Synth., synthesis; Attach., attachment; Modif., Modification; Meth., methylation; Oxid., oxidation; Red.,  reduction; Epim., epimerization. NA: not applicable. (f) Summary of capsule data on *C. jejuni* NCTC 11168 wild‐type and heptose mutants as per Wong et al. (2015). Plus (+) and minus (−) signs represent the presence or absence of the specified component, respectively: heptose and its methylation (OMe Hep), O‐phosphoramidate (MeOPN), Ethanolamine (N), 2‐amino‐2‐deoxyglycerol (NG), and the amounts of capsule produced by each mutant relatively to the wild‐type strain.

In Campylobacters, the structure and composition of the capsule are extremely diverse. A unique feature is the presence of modified heptoses with unusual ring configurations (D‐*altro‐*, L‐*gluco‐, ido‐)* (Aspinall et al., 1992, 1995; Chen et al., 2008; St Michael et al., 2002) and modifications such as C4/C6 dehydration, methylation or addition of an *O*‐methyl phosphoramidate (MeOPN) (McCallum et al., 2011). MeOPN can also be attached to other sugars of the capsule (McNally et al., 2007). The heptose can occupy different positions amongst various strains. For example, in *C. jejuni* strain NCTC 11168 which is the focus of this study, a 3,6‐OMe‐L‐*gluco*‐heptose branches off the capsule backbone (Michael et al., 2002) while in strain 81–176, a 6‐deoxy‐D‐*altro*‐heptose is part of the linear capsule units (Aspinall et al., 1992) (Figure 1a–d).

We pioneered the elucidation of the 3‐enzyme pathways (DdahA/B/C and MlghA/B/C) that lead to the production of GDP‐linked 6‐deoxy‐D‐*altro*‐heptose and 3,6‐OMe‐L‐*gluco*‐heptose that serve as precursors for incorporation of heptoses in the capsule of strains 81‐176 and NCTC 11168 (McCallum et al., 2011, 2012, 2013), and showed that the WcaG enzyme can reduce early heptose synthesis intermediates. WcaG was later shown to also fulfill the role of MlghA (Huddleston et al., 2020). These pathways share closely related epimerases (MlghB/DdahB) and reductases (MlghC/DdahC) that are responsible for strain‐specific heptose ring configurations that we and others studied at the biochemical and structural levels (Barnawi et al., 2021; Ghosh et al., 2022). They are present in most *C. jejuni* genomes but not in most other bacteria, and thus can represent new targets to prevent capsule‐mediated virulence.

### 1.4 Role of capsule and modified heptose in virulence of *C. jejuni* NCTC 11168

*C. jejuni* NCTC 11168 offers a unique opportunity to probe the contribution of the modified heptose to capsule function by knockout mutagenesis. This is because of the branching position of its heptose in the capsule that allows capsule assembly in the absence of heptose (Figure 1b). Heptose synthesis mutants in this strain are still encapsulated (Figure 1f) while heptose mutants in strains like 81–176 where the heptose is an integral part of the capsule chain (Figure 1a) are a‐capsular, and virulence phenotypes reflect the total loss of capsule and not the specific role of heptose.

To probe the heptose contribution to capsule function in *C. jejuni* NCTC 11168, we inactivated the *wcaG* (aka *mlghA*), *mlghB*, and *mlghC* genes mentioned above (Wong et al., 2015, and Figure 1e,f). We also generated a *wcaG*Δ mutant in which genes *cj1421c* to *cj1426c* are also deleted in addition to the *WcaG* gene disruption. As detailed in Figure 1e, these genes govern the synthesis and methylation of GDP‐*glycero‐manno*‐heptose, as well as the transfer of MeOPN to D‐α‐L‐*gluco‐*Hep*p* and β‐D‐Gal*f*NAc, respectively. The activity of these mutants was compared to that of a *kpsM* mutant which totally lacks capsule on its surface (Wong et al., 2015).

The heptose mutants exhibited higher sensitivity to killing by bile salts or serum, impaired invasion of human intestinal epithelial cells (*mlghB* and *mlghC* only) and decreased colonization of the chicken intestine compared to the wild‐type and even compared with the *kpsM* mutant (Wong et al., 2015). This demonstrates the role of the modified heptose in host colonization when the capsule is present. Differences of behavior observed across heptose mutants were attributed to variations in capsule amounts and in incorporation of modifications (MeOPN, ethanolamine, and 2‐amino‐2‐deoxyglycerol, Figure 1f) which possibly arose to compensate for the loss of the heptose functionality. Our transcriptional study excluded polarity effects downstream of the antibiotic cassettes used for gene inactivation but showed strong transcriptional effects in other areas of the capsular cluster (Wong et al., 2015), which can explain the variation of capsule composition in the mutants. Further, compensatory effects on other virulence components cannot be excluded, nor can the fact that free GDP‐heptose may have additional effects akin to the effects of free ADP‐heptose that is otherwise used for lipopolysaccharide (LPS) synthesis but affects virulence and host signaling in its free form (Malott et al., 2013). From the perspective of using the capsular heptose modification pathway as a target for intervention to eliminate *C. jejuni* or reduce its virulence, characterizing the global effects induced is essential, whether they are direct or not. This requires a multimodel and multispecies study considering the wide variations of the capsule roles observed across various models, host species, and *C. jejuni* strains.

### 1.5 Goals of our study and models used

We were interested in assessing interactions of *C. jejuni* NCTC 11168 with hosts that are relevant to its transmission to human beings, and in understanding the determinants of persistence in hosts where *C. jejuni* is a commensal versusGlutamine, a pathogen as this may provide strategies to better control its transmission and eliminate the bacteria. As campylobacteriosis is often tied to the consumption of contaminated chicken meat, and chicken flocks can be contaminated with *C. jejuni* via flies that serve as a bacterial vector (Hald et al., 2008; Royden et al., 2016), we focused on human, chicken and insect models. Specifically, we compared interactions between human THP‐1 and chicken Muquarrab Qureshi‐North Carolina State University (MQ‐NCSU) macrophages that are both responsive to *C. jejuni* (Jones et al., 2003; Qureshi et al., 1990), and between *Drosophila melanogaster* and *Galleria mellonella*, two insect models accepted to study host/bacteria interactions in the context of innate immunity (Browne et al., 2013; Hultmark, 2003; Igboin et al., 2012) and that represent a commensal and a pathogenic model, respectively. As macrophages in isolation do not recapitulate multifactorial in vivo interactions, the insect models afford a convenient approach to live animals and complement our prior chicken colonization studies (Wong et al., 2015). In each model, we investigated the fate of *C. jejuni* and the effect of *C. jejuni* on each host/host component, using the mutants described above to assess the role of capsule and heptose in all interactions. This extensive study highlights several mechanisms that contribute to species‐specificity, some being related to the capsule or its heptose modification pathway while others were not.

## 2 MATERIALS AND METHODS

### 2.1 Bacterial growth conditions

*C. jejuni* NCTC 11168 (ATCC 700819) freezer stocks were revived onto 5% sheep's blood trypticase soy agar (TSA) plates that contained 10 µg/mL vancomycin and 5 µg/mL trimethoprim as background antibiotics along with 15 µg/mL chloramphenicol for the *mlghB*, *mlghC*, *wcaG*, and *wcaG*∆ mutants and 30 µg/mL kanamycin for the *kpsM* mutant. Unless stated otherwise, all incubations were done at 37°C in microaerobic conditions (85% humidity, 10% CO_2_, and 5% O_2_). After overnight incubation, the bacteria were collected in 1 mL trypticase soy broth (TSB), and 100 µL adjusted to an OD_600_ of 0.01 were spread on TSA background plates and incubated overnight, as above. The bacteria were collected in 1 mL TSB and diluted to a specific OD_600_ dependent on the subsequent experiment. Heat‐inactivation was performed at 57°C for 45 min with intermittent shaking.

### 2.2 Biofilm assays

In a 100 mm diameter glass tube, 1 mL of a bacterial suspension adjusted to OD_600nm_ of 0.7 was incubated without shaking under aerobic or microaerobic conditions at 37°C for 4 days. A set of tubes was then taken out every day for 4 days, washed three times with water to remove planktonic cells, and left to dry for 30 min. Biofilm staining was done by adding 1 mL of 1% crystal violet and incubating at room temperature for 1 h. Excess stain was washed with water three times, and the tubes were left to dry for 30 min. Finally, the stained biofilms were detached from the glass wall by adding 1 mL of 30% acetic acid and incubating for 1 h. Two hundred microliters of each sample were transferred to a 96‐well microtiter plate to measure the absorbance at 590 nm (Reuter et al., 2010). Three replicates were prepared for each strain in three independent experiments each. Statistics were done using one‐way analysis of variance (ANOVA) test.

### 2.3 Nitrosative and H_2_O_2_ exposure assays

*C. jejuni* were grown as described above, harvested in the Brain Heart Infusion (BHI) broth and the suspension was normalized to an OD_600nm_ of 1 and 10 µL were deposited in wells of a 96‐well plate. For nitrosative stress, to promote the production of reactive nitrogen species, sodium nitrite was dissolved in BHI that had been pH‐adjusted to 5.0 (Gundogdu et al., 2011; Iovine et al., 2008) while H_2_O_2_ was prepared in BHI at pH 7.0. Serial dilutions of sodium nitrite or H_2_O_2_ (90 µL) were immediately added to the bacteria, which were incubated for 30 min under static microaerobic conditions at 37ᵒC. A minimum of three experiments were performed for each strain, each comprising triplicates with each concentration of sodium nitrite or of H_2_O_2_. After incubation, the bacteria were serially diluted 10‐fold (eight times) and plated in triplicate on TSB plates for colony forming unit (CFU) enumeration. CFUs were counted after 2 days of incubation.

### 2.4 *D. melanogaster* assays

*D. melanogaster* (wild‐type Canton‐S W118) were kindly provided by Dr. J. Burton (Western University, London, Ontario, Canada). The flies were raised at 26°C with 60% humidity on solid fly food made of 11.5 g/L of agar, 15 g/L of skim milk powder, 17.3 g of dried yeast, 73 g of corm meal, 76.6 mL of corn syrup and 5.8 mL of an acid mix made of 50 mL propionic acid and 3.2 mL phosphoric acid. To prepare synchronized flies, flies were incubated overnight on fly food, the flies were removed and the fly food containing new eggs was incubated 11 days till the eggs hatched. Newly hatched flies (20/*C. jejuni* strain and per biological replica) were transferred to fly food and incubated at 26°C for ~1.30 h while the *C. jejuni* bacteria were being prepared.

Separately, *C. jejuni* was grown as described above. Due to the presence of endogenous *C. jejuni* in the fly gut, all strains used comprised antibiotic resistance markers including a trackable wild‐type strain that was generated by inserting a kanamycin resistance cassette behind the c*j1319* gene which is remote from the capsular cluster under study and is functionally irrelevant (Zebian et al., 2016). All strains were resuspended at ~4.8E10 CFU/mL in 5% sucrose and 1 mL was used to soak a 2 cm^3^ cotton ball which was inserted in a tube that received 20 flies. After 21 h at 26°C, the flies were transferred onto regular fly food for 6 h. The viability of *C. jejuni* in the sucrose suspension was measured by CFU enumeration at time of fly exposure setup and of fly removal.

The flies were anesthetized by 3 min exposure to −20°C and dipped for 30 s in 70% ethanol. They were rinsed in water for 30 s and homogenized in quadruplets (five per strain) in 500 μL saline. The homogenate was spun down for 5 min at 1000 rpm to remove debris and eliminate intestinal bacteria without pelleting *C. jejuni*. This differential centrifugation step was omitted for the *kpsM* strain which aggregated and pelleted with all other components. The supernatant was used to enumerate *C. jejuni* by spreading 50 µL of serial dilutions onto Campylobacter selection media (Oxoid) supplemented with cefoperazone and strain‐specific selection (kanamycin or chloramphenicol) using glass beads. *C. jejuni* colonies were counted after 2 days of incubation.

### 2.5 *G. mellonella* assays

*G. mellonella* larvae (Recorp Inc.) were maintained on wood chips at 15°C. *C. jejuni* strains were grown as usual followed by resuspension at OD_600nm_ of 1 in 1.5 mL of Brucella broth supplemented with 0.25 mM sodium pyruvate and incubation at 37ᵒC for 4 h with shaking at 120 rpm. The cells were spun down gently and resuspended in 0.85% saline at an OD_600nm_ of 3. Larvae were infected with *C. jejuni* strains by microinjection (Hamilton syringe) of 10 µL in the right foreleg. The larvae were incubated at 37ᵒC and survival was recorded over 72 h of infection. Saline‐injected and noninjected controls were used. Batches of 15–20 larvae were injected for each strain. Considering the large number of strains to be tested, strains were split in overlapping groups, always including wild‐type and saline controls. The bacterial inoculum viability was measured by serial dilutions and CFU enumeration 2 days later.

### 2.6 Culturing MQ‐NCSU chicken macrophages

MQ‐NCSU cells were a kind gift from Dr S. Sharif (University of Guelph). Freezer stocks were made in 90% heat‐inactivated fetal bovine serum (FBS) (Multicell) and 10% dimethyl sulfoxide to a final concentration of ~2 × 10^6^ cells/mL. To begin culturing, 1 mL stocks were added to 6 mL of fresh MQ‐NCSU media (RPMI‐1640 containing 2.05 mM l‐Glutamine, 10% heat‐inactivated FBS, 100 units/mL penicillin, 100 µg/mL streptomycin, 2 µg/mL ciprofloxacin (for transcriptional studies only), 10 mM HEPES, and 10 µM β‐mercaptoethanol (BME, when needed to enhance adherence, not included for transcriptional studies), 1% nonessential amino acids [Gibco]). After centrifugation at 200–300 g for 5 min, the cell pellet was resuspended in 10 mL of MQ‐NCSU media and transferred to a T‐25 flask and incubated at 37°C under 5% CO_2_ for 1–2 days. MQ‐NCSU cells were maintained at concentrations of up to 1 × 10^6^ cells/mL, through passaging up to five times into T75 flasks seeded at 2 × 10^5^ cells/mL and media replacement every 2–3 days between passages. Adherent cells were recovered by treatment with 3 mL 0.25% trypsin‐EDTA (Multicell) for 5 min at 37°C and quenching with 4 mL of MQ‐NCSU media. Cells were plated 24 or 72 h before the start of an experiment at a concentration dependent on the assay being conducted (details below).

### 2.7 Culturing THP‐1 human monocytes and macrophages

THP‐1 macrophages (ATCC TIB‐202) were grown in RPMI‐1640 containing 2.05 mM l‐Glutamine, 10% heat‐inactivated FBS, 100 units/mL penicillin, 100 µg/mL streptomycin, 1 mM HEPES, 10 µM BME. T75 Flasks were seeded at ~2 × 10^5^ cells/mL and were incubated at 37°C in a 5% CO_2_ incubator for 1–2 days. Macrophages were maintained at concentrations between 2 × 10^5^ and 1 × 10^6^ cells/mL, via the addition of new media or through passage into T75 flasks up to five times.

After ~2 weeks of culturing, THP‐1 monocytes (nonadherent) were plated 24 h before the start of an experiment, or differentiated into THP‐1 macrophages, which were plated 72 h before the start of an experiment to accommodate for the differentiation treatment. Suspensions of THP‐1 cells in culture media to be differentiated were brought to a concentration of 250 nM phorbol‐myristate‐acetate (PMA). This suspension was then plated and incubated for 48 h before being washed twice with PBS and resuspended in antibiotic free media, for an additional 24‐h PMA‐free incubation. The final seeding density was dependent on the assay conducted.

### 2.8 Plating macrophages for lactate dehydrogenase release assay, Griess assay, TNF⍺ ELISA, and cytokine multiplex assay

MQ‐NCSU macrophages were plated at 6 × 10^4^ cells/well in a 96‐well plate 24 h before the start of coculture (allowing them to reach a target concentration of 8.5 × 10^5^ cells/well at start of coculture), while THP‐1 macrophages were plated at 8.5 × 10^5^ cells/well 72 h before the experiment. This was done because PMA‐treated THP‐1 cells lose much of their proliferative capability, and as such were plated at the target concentration.

The wells were washed twice with 150 µL PBS pH 7.4 before antibiotic‐free RPMI media was added. The appropriate volume of *C. jejuni* suspension at OD_600_ of 1 (equivalent to 1 × 10^9^ CFU/mL) was added to achieve a target MOI of 200 in a final volume of 275 µL/well. Some wells received 21 µL of 0.1 mg/mL  *Escherichia coli* VW187 LPS, media, or 0.45% triton X‐100. All samples were run in biological triplicates composed of technical triplicates. The interactions between *C. jejuni* and the macrophages were synchronized by centrifugation at 300 g for 2 min. The plates were incubated for various time points (indicated on figures) and two aliquots of each well were withdrawn for each assay: 50 µL for Griess assay, 35 µL for the LDH release assay, 115 µL for TNF⍺ ELISA and 115 µL for cytokine multiplex assay. All were frozen at −20°C for later use except the samples destined to LDH assay that were preserved at 4°C.

### 2.9 Griess assay

The Griess assay was performed according to Taha‐Abdelaziz et al. (2020) in 96‐well plates. A standard curve was made using serial dilutions of sodium nitrite (6.25–400 µM) in antibiotic‐free RPMI media. Controls without sodium nitrite were also included. Specifically, 50 µL of MQ‐NCSU macrophages culture supernatants or standard were mixed with 50 µL of 1% sulfanilamide prepared in 5% phosphoric acid. After 10‐min incubation at 26°C, 50 µL of 0.1% *N*‐1‐naphthyl ethylenediamine dihydrochloride (in water) were added. The OD_535nm_ was read after 10‐min incubation. The standard curve was fitted to the equation *Y* = (*A*–*D*)/(1 + (*X*/*C*)^*B*) + *D*, where *Y* is the OD_535nm_, *X* is the nitrite concentration in µM, and *A, B, C, and D* are modifiers adjusted to optimize the curve fit. Data were normalized to the average of the wild‐type samples at each time point.

### 2.10 LDH release assay

LDH concentrations were assessed using the Roche Cytotoxicity Detection Kit, following the manufacturer's instructions. Thirty‐five microliters of macrophage supernatant (stored at 4°C for up to 2 weeks) were diluted with 65 µL of 100 mM Tris pH 7.5. A standard curve was made using serial dilutions of LDH (Roche, 1.37 × 10^−3^ to 1 U/mL) made in 100 mM Tris pH 7.5. One hundred microliters of the kit reaction mixture were used per 100 µL of diluted supernatants or standard. Plates were incubated at 37°C for 30 min before reading the OD_490nm_. The data were fitted to the standard curve with the equation *Y* = (*A–D*)/(1 + (*X/C)^B*) + *D*, where *Y* is the OD_490nm_, *X* is the LDH concentration in U/mL, and *A, B, C, and D* are modifier adjusted to optimize the curve fit. Data were normalized to the average of triton‐containing samples (harvested after 5 min macrophage lysis, with maximal LDH release) at each time point.

### 2.11 TNF‐⍺ ELISA

Human TNF‐α quantification was conducted using the Human TNF‐α ELISA kit following the manufacturer's instructions (Ready Set Go kit, Fisher Scientific), using 100 µL of macrophage samples (diluted 4X in the provided diluent) or TNF‐α standard. The OD_450nm_ and OD_570 nm_ were recorded and the data were fitted to the standard curve using the equation *Y* = (*A–D*)/(1 + (*X/C*)^*B*) + *D*, where *Y* is the ∆OD_450‐570 nm_, *X* is the TNF‐α concentration in pg/mL, and *A, B, C, and D* are modifiers adjusted to optimize the curve fit. Data were normalized to the average value obtained with LPS (maximal activation) at each time point.

### 2.12 Cytokine multiplex

To detect the presence of human cytokines in THP‐1 macrophage supernatants after coculture with the capsular heptose mutants, samples were prepared as described above. For each of our three biological replica, supernatants from three wells were combined and 100 µL was set aside for analysis. Samples were processed by Eve Technologies to quantify the amount of interleukin (IL)‐1α, IL‐1β, IL‐6, IL‐8, IL‐10, IL‐12p40, IL‐18, and TNF‐α in solution, with two determinations per sample. Data were normalized to the average value obtained with LPS (maximal activation) at each time point.

### 2.13 Quantitative real‐time polymerase chain reaction (qRT‐PCR) analysis of cytokine production

Twenty‐four well plates were seeded with 7.5 × 10^5^ MQ‐NCSU cells or 1.25 × 10^6^ THP‐1 cells/well with a target of 1 × 10^6^ cells/well for both cell lines following the timelines and differentiation process as above. The cells were cocultured with *C. jejuni* (MOI of 200) for 0.5 to 3.5 h at 37°C. Lipopolysaccharide (from *E. coli* 0111:B4, Sigma) was used at a final concentration of 4 µg/mL. For optimization experiments, the THP‐1 cells were treated with 300 µL/well of Trizol Reagent (Thermo Fisher). For the full transcriptomics analysis of chicken macrophages, the cells were cocultured for 2 h 45 min and each well was treated with 300 µL Trizol, with three wells pooled for a total of 900 µL. Plates with Trizol were placed in the incubator for 5 min before cells were homogenized by pipetting up and down, and the lysates were stored at −80°C for later analysis. The viability of the inoculum was determined by CFU enumerations of spot‐plated serial dilutions.

For RNA extraction, the samples were treated with chloroform (60/300 µL trizol sample), the aqueous phase was recovered, and the RNA was precipitated with 100 µL of isopropanol. After centrifuging at 12,000 g for 15 min at 4°C, the RNA pellet was washed in 1 mL 75% ethanol and air dried for 5–10 min. The pellet was resuspended in 25 µL of DNAse‐ and RNAse‐free water, and the concentration of RNA was quantified using a NanoDrop 2000 spectrophotometer. Reverse transcription was done using SuperScript II reverse transcriptase (Invitrogen) following the manufacturer's instructions, using either 1 µg of THP‐1 RNA or 3 µg of MQ‐NCSU RNA and using random hexamer primers.

Quantitative PCR (qPCR) was performed using 5 µL of PowerUp SYBR green master mix (Applied Biosystems), 0.2 µL of each gene‐specific primer at 25 µM (Table 1), 1–2 µL of cDNA (Not diluted for THP‐1, diluted 1:10 for MQ‐NCSU) for a final volume of 10 µL. Samples were incubated at 50°C for 2 min and 95°C for 2 min in a Corbett Rotorgene 6000 thermocyler, followed by 40 cycles of 95°C for 15 s, 56°C for 15 s, and 72°C for 1 min. For optimization work, the fold changes were calculated as 2^‐∆∆*Ct*^ where ∆∆*Ct* = average ∆Ct (wild‐type coculture samples)—average ∆*Ct* (untreated samples). In turn, ∆*Ct* = *Ct* (gene of interest)—Ct (housekeeping gene). For final transcriptomics analysis, the fold change was calculated using the Pfaffl equation which accounts for primer efficiencies (Pfaffl, 2001). In that case, the fold change was calculated as Efficiency(test gene) –∆*Ct* (test gene)/efficiency (housekeeping gene) –∆*Ct* (housekeeping gene) where ∆*Ct* (test gene) = average *Ct* (test gene in coculture samples)—average *Ct* (test gene in untreated samples) and ∆*Ct* (housekeeping gene) = average *Ct* (housekeeping gene in coculture samples)—average *Ct* (housekeeping gene in untreated samples). Primer efficiencies were calculated using the LineRegPCR software.

**Table 1:** Gene‐specific primers used during qPCR on cDNA generated from human and chicken macrophages.

Gene	F/R	Sequence	Size	TM (°C)	Source
**Human**					
TNF‐α	F	TCAGCAAGGACAGCAGAGG	124	60	Yang et al. (2016)
	R	GTATGTGAGAGGAAGAGAACC		62	
Interleukin (IL)‐1β	F	ATGGCTTATTACAGTGGCAATG	138	62	Yang et al. (2016)
	R	GTAGTGGTGGTCGGAGATTC		62	
IL‐6	F	GTAGTGAGGAACAAGCCAGAG	240	64	Yang et al. (2016)
	R	CATGCTACATTTGCCGAAGAG		62	
IL‐8	F	AACCATCTCACTGTGTGTAAAC	66	62	Harrison et al. (2005)
	R	ATCAGGAAGGCTGCCAAGAG		62	
Il‐10	F	GAGGCTACGGCGCTGTCAT	60	62	Kim et al. (2018)
	R	TGCTCCACGGCCTTGCTC		60	
GAPDH	F	CAACGGATTTGGTCGTATTGG	72	62	Harrison et al. (2005)
	R	GCAACAATATCCACTTTACCAG		64	
**Chicken**					
IL‐1β	F	TGAGGCTCAACATTGCGCTG	213	62	Quinteiro‐Filho et al. (2015)
	R	TGTCCAGGCGGTAGAAGATG		62	
IL‐6	F	CGTGTGCGAGAACAGCATGG	107	64	Quinteiro‐Filho et al. (2015)
	R	GGCATTTCTCCTCGTCGAAG		62	
IL‐8	F	CCAAGCACACCTCTCTTCCA	176	62	Quinteiro‐Filho et al. (2015)
	R	GCAAGGTAGGACGCTGGTAA		62	
Il‐10	F	CTTTGGCTGCCAGTCTGTGT	221	62	Taha‐Abdelaziz et al. (2020)
	R	TCATCCATCTTCTCGAACGTC		62	
GAPDH	F	GCCGTCCTCTCTGGCAAAG	72	62	Vaezirad et al. (2017)
	R	GTAAACCATGTAGTTCAGATCG		62	
iNOS	F	CAGCAGCGTCTCTATGACTTG	182	64	Taha‐Abdelaziz et al. (2020)
	R	ACTTTAGGCTGCCCAGGTTG		62	
RPL37a	F	ATTGAAATCAGCCAACACGC	94	58	*
	R	AGGACCCACAGTGCCAGATAC		68	

*Note*: F/R stands for forward and reverse. Size refers to the amplicon size. TM is the melting temperature that was determined using GeneRunner version 6.5.52, representing the combined GC + AT melting temperature. Sources indicate publications where original primer sequences were identified. Some sequences were modified to increase the consistency of annealing temperature or to minimize the formation of internal loops, by adding or removing complementary bases from the 5′ and 3′ ends. Primers marked with * were designed using NCBI primer blast, specifying primers must span one exon–exon junction.

### 2.14 Reactive oxygen species fluorescence assay

To assess the induction of reactive oxygen species within the host cells, 96 well plates were seeded with 6 × 10^4^ MQ‐NCSU cells/well 24 h before the start of coculture, or 1.6 × 10^5^ THP‐1 cells/well 72 h before the start of coculture, to have 8.5 × 10^4^ cell/well for both macrophages at coculture time. THP‐1 cells were differentiated into monocytes using PMA as described above. Wells with macrophages were washed twice with PBS pH 7.4 before being incubated in PBS pH 7.4 containing 50 µM 2′,7′‐dichlorofluorescein diacetate (DCF) (Sigma) at 37°C (5% CO_2_) for 30 min. Wells were again washed twice before adding 100 µL of *C. jejuni* suspended at 1.7 × 10^8^ cells/mL in antibiotic‐free RPMI devoid of phenol red (Dpr‐RPMI, Wisent), or of LPS (7.5 µg/mL in Dpr‐RPMI) or Dpr‐RPMI media alone. A few wells with macrophages were not treated with DCF to obtain readings of background fluorescence. After 30 or 90 min of incubation, 75 µL of supernatant was taken for fluorescence reading (BioTek Cytation 5) with excitation at 485 nm and emission at 528 nm. Data were normalized to the average value obtained with LPS (maximal activation) at each time point.

### 2.15 Adhesion, uptake, and intracellular survival assays

MQ‐NCSU macrophages were seeded at a density of 2.5 × 10^5^ cells/well 24 h before the start of coculture (500 µL/well in 24 well plates). As both THP‐1 macrophages and monocytes were used in this assay, THP‐1 macrophages (cells treated with PMA) were seeded at 2.5 × 10^5^ cells/well 72 h before the start of coculture, while monocytes were seeded at 1 × 10^5^ cells/well at the same time point (500 µL/well in 24 well plates). Before the start of coculture, all wells were pelleted at 300 g for 5 min and washed twice with PBS pH 7.4 (PBS). Five hundred microliters of OD_600nm_ of 0.01 bacterial suspensions made in antibiotic‐free macrophage media were added to achieve an MOI of 100, and the plates were centrifuged at 300 g for 2 min before incubation. The viability of the inoculum was determined by CFU enumeration after spot‐platting serial dilutions. Data were normalized to the wild‐type average at each time point tested.

To determine adherence to host cells, the incubation was performed at 4°C for 30 min. Control samples were taken to enumerate the bacteria in suspension and the wells were washed three times with cold PBS, with centrifugation at 300 g for 5 min before each wash to prevent the loss of nonadherent monocytes. After washing, 200 µL of ddH_2_0 was added to the wells, and the plate was incubated at 37°C for 20 min to lyse the host cells. After pipetting up and down to disrupt the macrophages/monocytes, the lysate was serially diluted in saline and 10 µL of each dilution was spot‐plated on background TSA plates to enumerate the adherent bacteria by CFU counting.

To determine bacterial uptake, the incubation was performed at 37°C for 1 or 3 h. Samples were taken to enumerate the bacteria in suspension, excess *C. jejuni* was removed with one wash with PBS as above. The cells were then incubated with 500 µL RPMI containing 200 μg/mL gentamicin for 1 h at 37°C to kill all extracellular bacteria. Wells were then washed three times with PBS as above and incubated with 200 µL of ddH_2_O at 37°C for 20 min to lyse the host cells. The lysate was serially diluted at two‐fold intervals and 100 µL was spread onto background TSA plates to enumerate the intracellular bacteria.

To determine the intracellular survival, the incubation was performed at 37°C for 2 h. The excess *C. jejuni* was removed with one wash of PBS. The samples were treated with gentamicin for 1 h (as above), washed three times with PBS, and resuspended in antibiotic‐free media for further incubation for 1 or 3 h. The cells were then washed, lysed, and enumerated by spread‐plating as above.

## 3 RESULTS

### 3.1 The capsule and its modified heptose support biofilm growth in aerobic conditions

**Figure 2:**
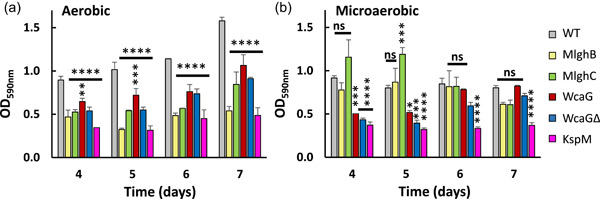
Biofilm formation by  *Campylobacter jejuni* NCTC 11168 wild type and mutants under aerobic (a) and microaerobic (b) conditions. Biofilm formation was measured every day for 4 days after the initial 4‐day incubation where biofilms become sturdy enough to allow accurate quantitation. Statistics were done using two‐way analysis of variance within each data set. *p < 0.05, **p < 0.01, ***p < 0.001, and ****p < 0.0001.

It was shown previously for *C. jejuni* that biofilm formation increases under aerobic conditions (Reuter et al., 2010) and that the capsule plays a role in biofilm formation (Wang et al., 2015). We assessed the role of the capsule and its heptose in biofilm formation in strain NCTC 11168 under both aerobic and microaerobic conditions. The wild‐type formed similar amounts of biofilm under both conditions during the initial 4‐day incubation (Figure 2) but biofilm formation continued throughout the 7th day under aerobic conditions while it stopped under microaerobic conditions. This suggests that growth as a biofilm may be triggered by oxygen exposure. In contrast, the capsule‐less *kspM* mutant made very little biofilm, and the amount did not increase over time in either condition. This indicates that the capsule is important for biofilm establishment in this strain.

All heptose mutants made significantly less biofilm than the wild‐type in aerobic conditions after 4 days. This suggests that the heptose modification pathway is important for maximal biofilm formation under aerobic conditions though it is not essential. Under microaerobic conditions, the *mlghB* and *mlghC* mutants showed no defect, and a slightly faster kinetic of biofilm formation was even observed for the *mlghC* mutant, though the biofilms appeared less stable than wild‐type biofilms as less biofilm was recovered upon prolonged incubation. The *wcaG* and *wcaGΔ* mutants behaved opposite from the *mlghB* and *mlghC* mutants, being impaired in the initial phase of biofilm establishment (till Day 5) but reaching wild‐type levels afterward. The altered kinetics and amounts of biofilm formation indicate a role of the heptose modification pathway in biofilm stabilization. Moreover, the similar patterns observed between the *wcaG* and *wcaGΔ* mutants indicate that the phosphoramidate capsule modification that is lacking in the *wcaGΔ* mutant does not play a major role in the process.

### 3.2 The capsule and its modified heptose contribute to the survival of *C. jejuni* in the fly gastrointestinal track

Flies serve as vectors mediating contamination of chicken flocks by *C. jejuni* (Hald et al., 2008; Royden et al., 2016), suggesting that *C. jejuni* can survive transit in their gastrointestinal (GI) tract, where it will encounter innate immune components, including macrophages and their redox effectors. The Drosophila GI model is relevant to *C. jejuni* which occupies a GI niche in all hosts. All *C. jejuni* recovered in this model originates from the fly gut since the flies are sterilized in ethanol before homogenization for *C. jejuni* enumeration.

**Figure 3:**
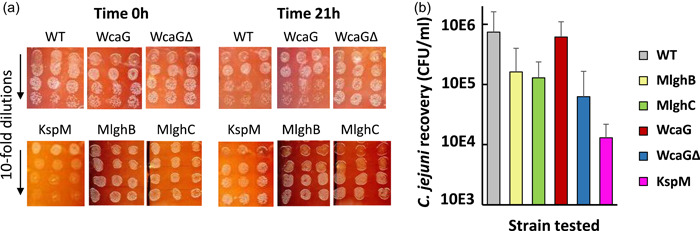
Role of the capsule and its modified heptose in the ability of  *Campylobacter jejuni* to survive passage through the gastrointestinal tract of *Drosophila melanogaster*. Aged‐matched drosophila were fed for 21 h on a sucrose solution containing 1.5E10 CFU/mL of *C. jejuni* or none. The flies were returned to solid food for 6 h before being sterilized and homogenized as quadruplets for enumeration of live *C. jejuni* by spot‐plating on selection media. (a) The viability of the inoculum at the time of preparation and after 21 h in the fly incubator showed that all strains had similar viability at the time of inoculation and the end of fly feeding. (b) The concentration of the fly homogenate in live *C. jejuni* that have survived passage through the fly gastrointestinal tract. For each strain, three biological replicates were conducted, with five quadruplets of flies (20 flies/experiment). The limit of detection of the assay was 10E3 CFU/mL. Statistics done on Prism by two‐way analysis of variance with Tukey's multiple comparison tests and by *T*‐test showed no significance though a downward trend was seen for most mutants.

**Figure A1:**
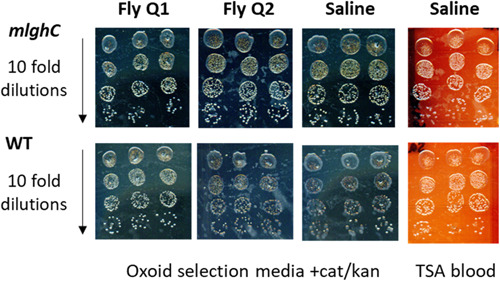
Oxoid selection media coupled to kanamycin or chloramphenicol selection is suitable to recover our *Campylobacter jejuni*  inoculum even in the presence of fly homogenate. Our wild‐type (WT) in which we introduced a kanamycin (kan) resistance cassette and our *mlghC* mutant that is chloramphenicol (cat) resistant were recovered in full when prepared in saline and spot plated on Oxoid Campylobacter selection media supplemented with appropriate antibiotics, showing equal growth as on standard TSA blood plates. The presence of fly homogenate prepared from four flies (quadruplets Q1 and Q2) in ~300 μL saline had no negative effect on the recovery of *C. jejuni* either. It also did not lead to enhanced bacterial counts, which indicates that endogenous *C. jejuni* was eliminated by the antibiotic selection.

To use this model, we ascertained that all our strains were equally resistant to the Campylobacter selection media (Examples in Figure A1) and that our antibiotic selection eliminated all endogenous *C. jejuni* from fly homogenates. We also ascertained that innate defense components (reactive oxygen species [ROS], antimicrobial peptides…) which may not be present in the fly gut but may be released from the homogenization of whole flies would have no deleterious effects on the survival of *C. jejuni* (Examples on Figure A1). Finally, since flies fed on *C. jejuni* resuspended in sucrose for 21 h at 26°C in aerobic conditions that are optimal for flies but suboptimal for *C. jejuni* (Levin, 2007), we ascertained that this incubation had no impact on the viability of our strains (Figure 3a). All mutants were then tested using inoculum of comparable viability (Figure 3a). Recovery of wild‐type *C. jejuni* at ~10E6 CFU/ml of fly homogenate indicated that it was readily ingested by the flies and survived in the GI tract at least for 6 h after the original inoculum was taken away from the flies (Figure 3b). Ingestion of *C. jejuni* did not affect the viability of the flies in this time frame, in line with its commensal nature in flies. A clear trend toward decreased survival of the a‐capsular *kpsM* mutant compared with the wild‐type was noted, with a 2‐log difference (Figure 3b). Three of the heptose mutants also showed a similar trend. While no statistical significance was seen, the trends suggest that the whole capsule affords protection to *C. jejuni* in the fly gut and that the modified heptose contributes to this, although moderately. The MeOPN also appears to be important for survival in the fly gut in the context of heptose‐less mutants based on the decreased survival of the *wcaGΔ* mutant (no heptose, no MeOPN) compared with the *wcaG* mutant (no heptose, with MeOPN).

### 3.3 The capsule and its heptose limit the virulence of *C. jejuni* toward *G. mellonella* larvae

**Figure 4:**
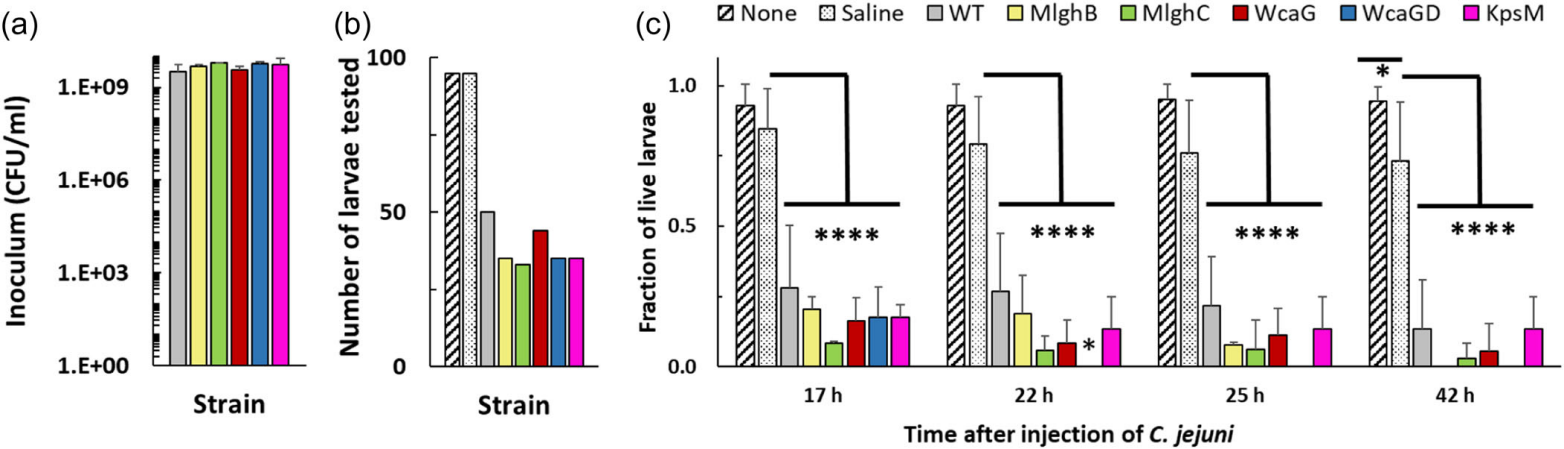
Effect of  *Campylobacter jejuni* and capsular mutants on the survival of *Galleria mellonella*. *G. mellonella* larvae were injected with 10 µL of *C. jejuni* at ~5E9 CFU/mL or saline and viability of the larvae was monitored over 42 h at 30°C. A “none” control where larvae were not injected at all served as quality control for the injection technique. (a) Viability of the inoculum of the various *C. jejuni* strains used. (b) Total number of larvae tested for each condition. Biological replicates comprising 10–25 larvae per strain were conducted with overlapping subsets of *C. jejuni* strains and always comprised the controls, resulting in *n* = 8 for saline and “none” controls and *n* = 3 for all *C. jejuni* strains. (c) Fraction of larvae surviving treatment with various strains of *C. jejuni* or with controls at each time point. Statistics were done on Prism by two‐way analysis of variance with Tukey's multiple comparison tests. *p < 0.05, ****p < 0.0001.

In the *G. mellonella* larvae model, bacteria are injected into the foreleg septum, resulting in a septic infection that can kill the larvae. Batches of 15–20 larvae were injected with ~5 × 10E9 CFU/mL of *C. jejuni* for each experiment (Figure 4a). Strains were split into overlapping groups always including “no‐injection” and saline controls, resulting in more data for the controls than for the test strains (Figure 4b). The controls demonstrate that the injection process did not adversely affect the larvae up to 25 h after injection (Figure 4c). However, injection of wild‐type *C. jejuni* led to a drastic reduction of larvae viability as early as 17 h after injection, with very few larvae surviving by 42 h (Figure 4c). This established the pathogenic character of *C. jejuni* in this model. The a‐capsular *kpsM* mutant and all heptose modification mutants showed strong killing abilities, and surprisingly higher potency than the wild‐type, indicating that the capsule and heptose are not necessary for pathogenicity in this model, though no statistical significance was obtained except for *wcaGΔ* vs wild‐type at 22 h (*p* = 0.0308).

### 3.4 *C. jejuni* adherence to, uptake by, and intracellular survival in macrophages are affected in a species‐ and mutant‐specific manner

**Figure 5:**
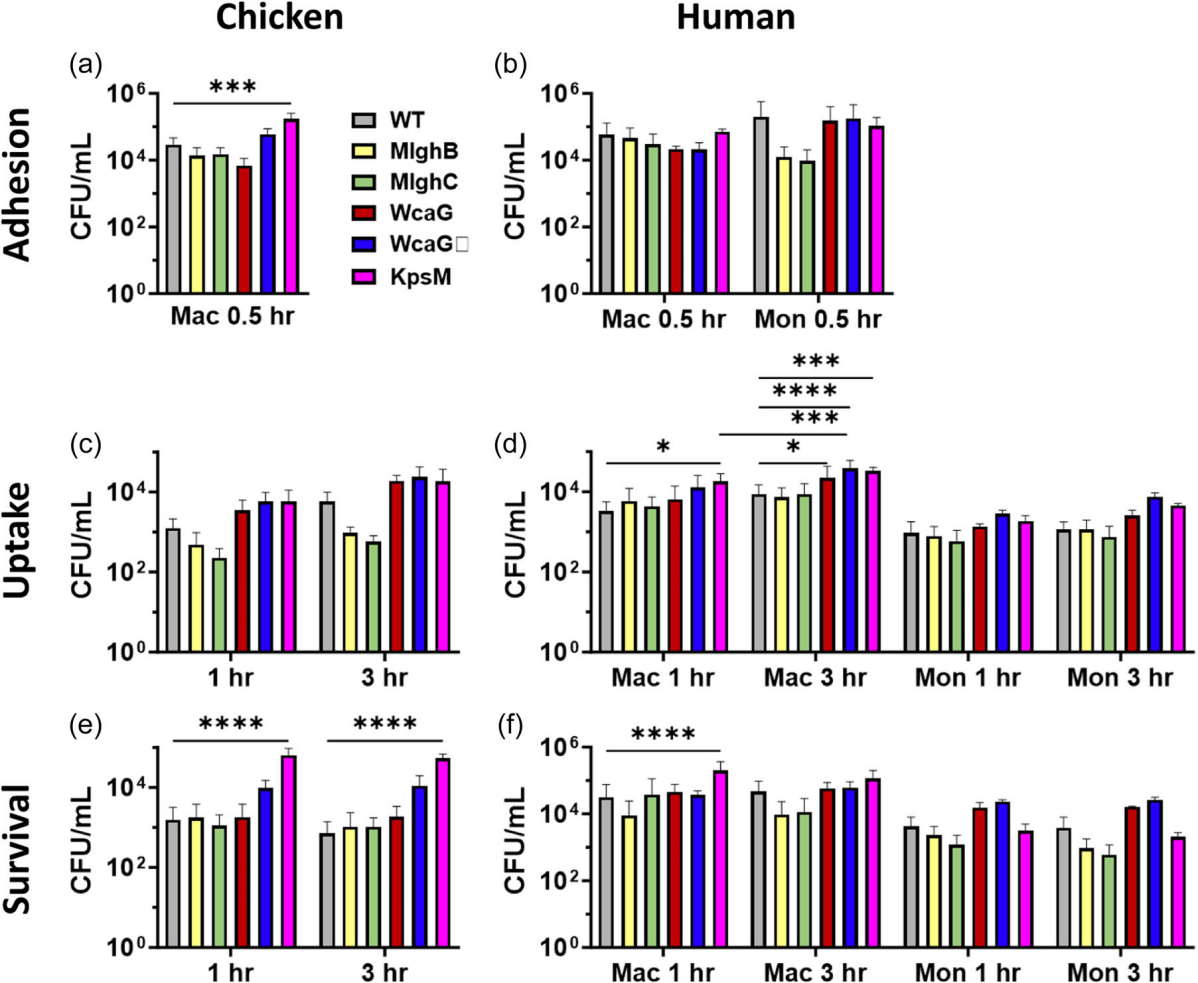
Interactions of  *Campylobacter jejuni* capsular heptose mutants to chicken and human macrophage (Mac) and/or monocytes (Mon). Inoculum and culture controls are provided in Figures A2 and A3. Panels a, c, and e for chicken MQ‐NCSU macrophages and (b, d, and f) for human cells. (a, b) Adhesion measured after 30 min of coculture at 4°C, with 3–4 biological replicates for mutants and 6–8 replicates for wild‐type. (c, d) Bacterial uptake measured after 1 or 3 h of coculture and elimination of external bacteria by gentamicin treatment before bacterial enumeration. Number of biological replicates: three for chicken studies, 3–5 for mutants, and eight for wild‐type for human macrophages, 2–3 for mutants, and six for wild‐type for human monocytes. (e, f) Intracellular survival was measured by allowing uptake for 2 h before gentamicin treatment and pursuing the incubation for 1 or 3 h before bacterial enumeration. The number of biological replicates: three for mutants and six for wild‐type for chicken studies, 2–4 for mutants and nine for wild‐type for human macrophages, 2–3 for mutants, and seven for wild‐type for human monocytes. Error bars represent the standard deviation across biological replicates. Statistical analysis was conducted by analysis of variance and subsequent Dunnett tests to compare wild‐type and mutants and Bonferroni tests to compare outputs between time points. ND, not determined. *p < 0.05, ***p < 0.001, ****p < 0.0001.

**Figure A2:**
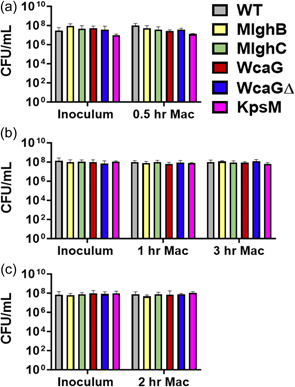
Viability of the inoculum used for interaction studies between *Campylobacter jejuni* and chicken macrophages. (a) For adherence studies. (b) For uptake studies. (c) For intracellular studies. In each case, the inoculum was enumerated by CFU counting at the time of coculture setup, and at the time of removal of the nonadhered or external bacteria, as applicable. Mac: macrophages. The adherence, uptake, and intracellular data are presented in Figure 5.

**Figure A5:**
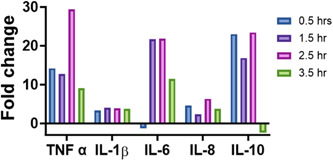
Preliminary quantitative real‐time polymerase chain reaction optimization on human macrophages. The data show induction of transcription of each cytokine tested, with maximal amounts seen at 2.5 h of coculture for all (*N* = 1).

In all macrophage assays, the viability of the inoculum was comparable across strains, and samples taken at various times indicated that coculture conditions did not affect bacterial viability during the interactions (Figures A2, A5). All strains could adhere to, enter in, and survive in all cell types, however species‐specific and mutant‐specific effects were noted (Figure 5).

In chicken macrophages (Figure 5a,c,e), uptake of wild‐type *C. jejuni* was slow and limited despite abundant adherence, and intracellular survival was limited as indicated by the lack of increase of bacterial counts between the 1 and 3 h time points despite the increase in uptake. The capsule mitigated each step of the interaction, since higher than wild‐type counts were obtained for the *kspM* mutant, with significance for adhesion and intracellular survival. The *wcaGΔ* showed a similar trend, while the *wcaG* mutant only showed elevated uptake levels. This suggests a role of *wcaG* and the MeOPN modification genes in mitigating uptake, while the MeOPN modification also mitigates adherence and intracellular survival when present in a heptose‐less mutant. The *mlghB* and *mlghC* mutants behaved differently, showing wild‐type‐like adherence and intracellular recovery but a reduced uptake. This suggests that their intracellular survival is enhanced, allowing accumulation despite the limited uptake.

In human macrophages (Figure 5b,d,f), strong adherence of wild‐type *C. jejuni* was observed at similar levels as in chicken macrophages, and uptake by and survival within human macrophages appeared more efficient relative to the original adherence levels, especially considering the increase in bacterial counts after original uptake. The capsule contributed to mitigating uptake and intracellular survival but did not affect adherence. The *wcaG* and *wcaGΔ* mutants showed wild‐type‐like adherence and intracellular recovery despite an enhanced uptake (at 3 h). This suggests that their intracellular survival is slightly impaired, and thus *wcaG* and the MeOPN modification support intracellular survival. The *mlghB* and *mlghC* mutants showed wild‐type‐like properties for adherence and uptake but a trend for reduced long‐term intracellular survival.

Finally, in human monocytes, as expected for nondifferentiated cells, limited uptake was observed despite strong adherence of wild‐type *C. jejuni*, resulting in low though steady intracellular survival counts. The capsule‐less mutant had wild‐type‐like adherence and survival and slightly elevated uptake suggesting the capsule limits the uptake slightly. The *wcaG* and *wcagΔ* mutants showed a trend toward elevated uptake and survival, indicating mitigation of these steps by *wcaG* and the MeOPN modification. The *mlghB* and *mlghC* mutants stood apart, with much‐reduced adherence (1.5 log less than wild‐type) that could still be counterbalanced by efficient uptake to reach wild‐type level, but much impaired intracellular survival of the internalized bacteria. Thus, the *mlghB* and *mlghC* genes (and their activity) support adherence and intracellular survival in the wild‐type context while mitigating uptake.

### 3.5 *C. jejuni* does not confer significant cytotoxicity to host macrophages under the conditions tested

**Figure 6:**
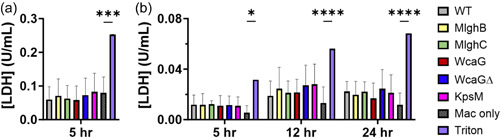
Minimal host macrophage cytotoxicity induced by *Campylobacter jejuni* wild‐type and mutants. (a) MQ‐NCSU macrophages (*n* = 3), (b) phorbol‐myristate‐acetate (PMA)‐treated THP‐1 macrophages (*n* = 3–4). Supernatants from macrophage (Mac) cocultures with *C. jejuni* were assessed for the presence of LDH. Triton was used at 0.45%. Statistical analysis was conducted by one‐way analysis of variance (ANAVO) and subsequent Dunnett tests for MQ‐NCSU cells. Analysis was conducted by two‐way ANOVA with subsequent Dunnett tests for THP‐1 cells. Each biological replica is comprised of three technical replicates. Error bars represent the standard deviation, all comparisons are to macrophage only. *p < 0.05, ***p < 0.001, ****p < 0.0001.

The lactate dehydrogenase (LDH) release assay data indicate that *C. jejuni* (wild‐type or mutant) does not significantly induce cytotoxicity of either macrophage species (Figure 6). The levels of released LDH remained small in comparison to the total amount of LDH present in these cells (determined by lysing the cells with triton) and no differences were observed between wild‐type and mutants. Overall, cytotoxicity is minimal and would not differentially affect the induction of effectors by wild‐type *C. jejuni* or the mutants for either cell line.

### 3.6 The *C. jejuni* NCTC 11168 capsular heptose diminishes the capsule‐induced nitrite induction in chicken macrophages

**Figure 7:**
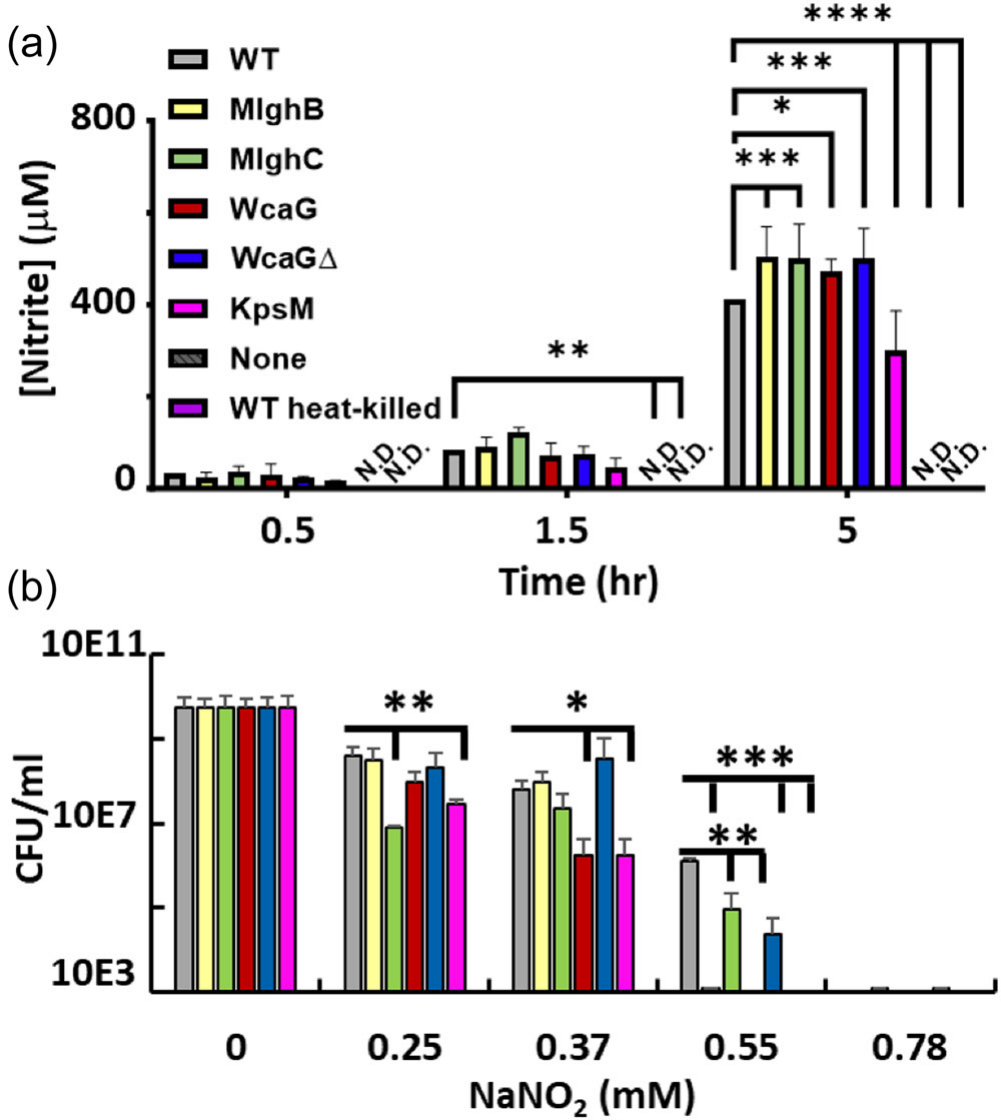
Ability of  *Campylobacter jejuni*  wild‐type and capsular heptose mutants to elicit nitrite induction in MQ‐NCSU macrophages and to survive nitrosative stress. (a) Nitrite induction measured from coculture supernatants at 30 min (*n* = 3), 90 min (*n* = 3), and 5 h of coculture (*n* = 6 for wild‐type and *n* = 3 for all others) via Griess assay. Analysis was conducted by two‐way analysis of variance (ANOVA) with subsequent Dunnett tests. ND, none detected. (b) Nitrosative stress data normalized to the untreated wild‐type control. Unnormalized data presented in Figure A4a. When no bacteria were recovered, the value was assigned to the detection limit of this assay (10E3 CFU/mL). Data from 3 to 12 biological replicates depending on the strains and the concentrations tested. No significance observed by ANOVA. Significance obtained by *T*‐tests relative to wild‐type at each NaNO_2_ concentration. For both panels, the error bars represent the standard deviation, and all comparisons are to wild‐type with *p < 0.05, **p < 0.01, ***p < 0.001, and ****p < 0.0001.

In the human macrophages, nitrite levels were undetectable or only slightly above the level of detection of the Greiss assay, independent of capsule and/or heptose expression. This is in line with other reports that THP‐1 macrophages do not produce detectable NO upon stimulation (Cinelli et al., 2020; Fontán et al., 2008; Pérez‐Pérez et al., 1995). In chicken macrophages, the wild‐type *C. jejuni* caused significant NO induction after 90 min and 5 h of coculture (Figure 7a), as also shown by others (Brisbin et al., 2015; Shojadoost et al., 2019; Taha‐Abdelaziz et al., 2020), and the induction was specific to live bacteria. The a‐capsular *kspM* mutant had diminished levels of nitrite induction relative to the wild‐type while the modified heptose mutants all had significantly elevated levels at the 5‐h time point.

Overall, this indicates that NO induction by MQ‐NCSU macrophages in response to *C. jejuni* NCTC 11168 is a relatively slow process that is dependent on the viability of the bacteria and is enhanced by the presence of the capsule. The capsular heptose pathway acts to dampen nitrite induction caused by live *C. jejuni*.

### 3.7 The capsule and its modified heptose limit the sensitivity of *C. jejuni* to nitrosative stress

To investigate the relevance of the NO burst modulation by capsule and heptose reported above to *C. jejuni* survival, we tested the sensitivity of wild‐type and mutants to nitrosative stress in vitro. Nitrosative stress was deleterious to all strains tested with decreased bacterial survival as the NaNO_2_ concentration increased (Figure 7b) and a lack of recovery of any bacteria at 0.78 mM NaNO_2_. The lack of phosphoramidate in the capsule of the *wcaGΔ* mutant seems to increase the threshold of resistance to nitrosative stress relative to the *wcaG* mutant. The capsule played a protective role against nitrosative damage as evidenced by lower than wild‐type recovery of the *kspM* mutant at all concentrations while the heptose modification mutants were more sensitive than wild‐type, with statistical significance when the concentration reached 0.55 mM. This indicates that when the capsule is present, the heptose contributes to the protective effect of the capsule against nitrosative stress.

### 3.8 Live *C. jejuni* NCTC 11168 diminishes the levels of ROS produced by host macrophages independently from the capsule or its heptose

**Figure 8:**
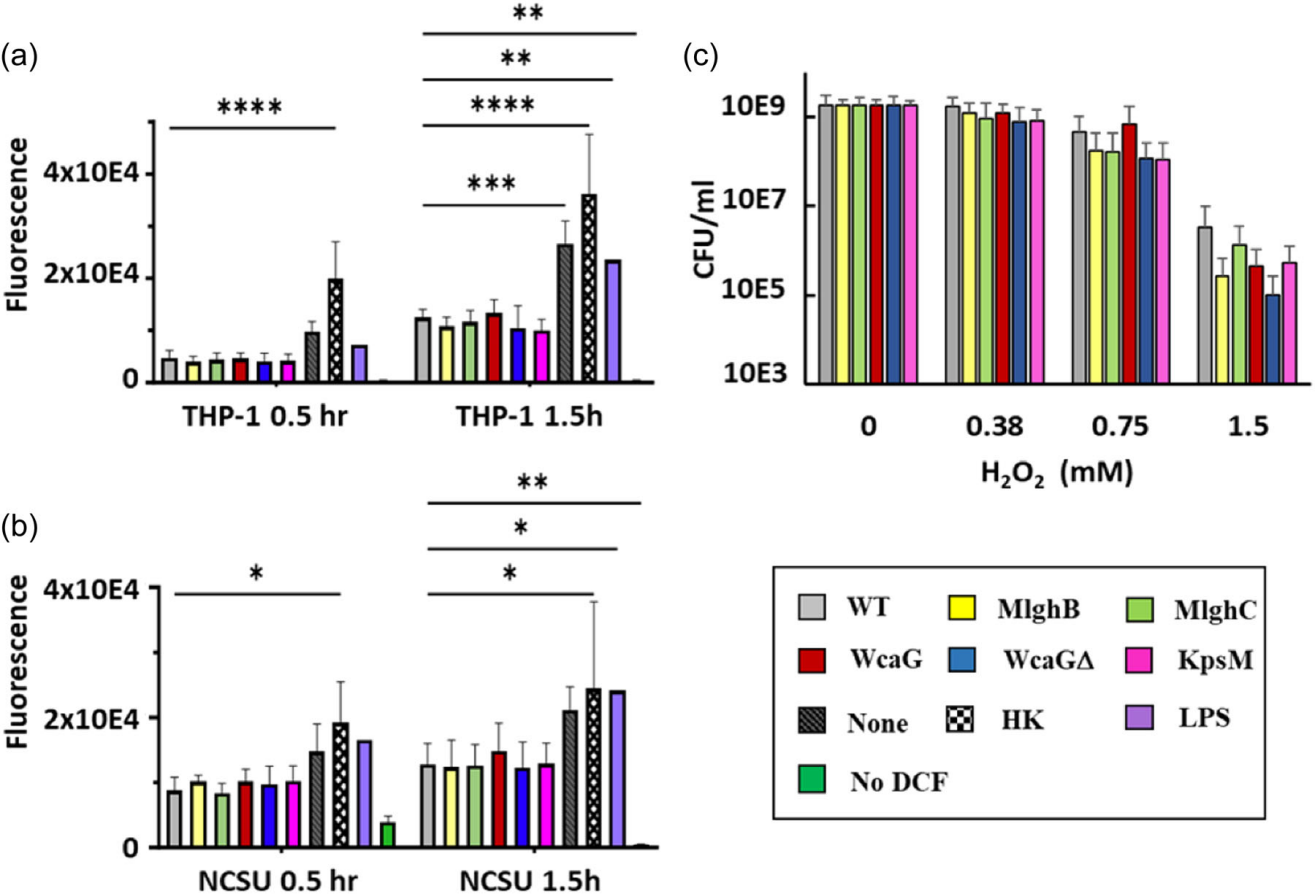
Induction of reactive oxygen species in THP‐1 human and MQ‐NCSU chicken macrophage by  *Campylobacter jejuni* and sensitivity of *C. jejuni* to oxidative stress. (a, b) ROS induction in human THP‐1 and chicken NCSU macrophages. The macrophages were incubated with 2′,7′‐dichlorodihydrofluorescein diacetate (DCF) for 30 min before challenge with *C. jejuni* or controls. The fluorescence of the culture supernatant was measured with emission at 485 nm and excitation at 528 nm. Error bars represent standard deviation from three biological replicates each comprised of three technical replicates. Analysis was conducted by two‐way analysis of variance (ANOVA) with subsequent Dunnett tests, with *p < 0.05, **p < 0.01, ***p < 0.001, ****p < 0.0001. (C) Sensitivity to H_2_O_2_ recorded as CFU/mL of bacteria recovered after 30 min of exposure in microaerobic conditions. Data are normalized to the unexposed wild type. The nonnormalized data are presented in Figure S4a. No statistical significance was observed by two‐way ANOVA or *T*‐test.

In both macrophage cell lines, coculture with wild‐type *C. jejuni* caused a marked reduction in ROS levels compared with the negative control (“none”) at both times tested (30 and 90 min) though statistical significance was only reached for THP‐1 cultures at 90 min (Figure 8a,b). This reduction was also observed upon coculture with the a‐capsular mutant or with the heptose mutants. Interestingly, this ROS quenching phenotype was specific to live *C. jejuni*, as the heat‐killed wild‐type caused slightly higher levels than the negative control and than live wild‐type across all cell lines and time points tested (Figure 8a,b). This quenching was not recapitulated by the addition of LPS which did not induce ROS beyond the level of the negative control.

Overall, the data indicate that *C. jejuni* NCTC 11168 does not trigger the expected ROS burst and is also able to diminish the background induction of ROS by host cells from both species and that this function is independent of the capsule and its heptose but requires live bacteria.

### 3.9 The capsule and heptose only provide limited protection against oxidative stress

The macrophage data showing an active dampening of macrophage‐mediated ROS burst suggest that *C. jejuni* is sensitive to oxidative stress and may have evolved a capsule‐ and heptose‐independent mechanism to avoid exposure to ROS. Surprisingly, when *C. jejuni*'s sensitivity to oxidative stress was assessed using exposure to H_2_O_2_, all strains were fairly resistant up to 0.75 mM H_2_O_2_, after which 2–4 log of viability were lost for all strains. The wild‐type seemed slightly more resistant than the mutants at the highest concentration tested, though statistical significance was not reached (Figure 8c). This suggests that the capsule and its heptose only provide limited protection against H_2_O_2_ exposure.

### 3.10 *C. jejuni* NCTC 11168 induces the production of many cytokines in human macrophages, but its capsule and heptose are not involved in the process

**Figure 9:**
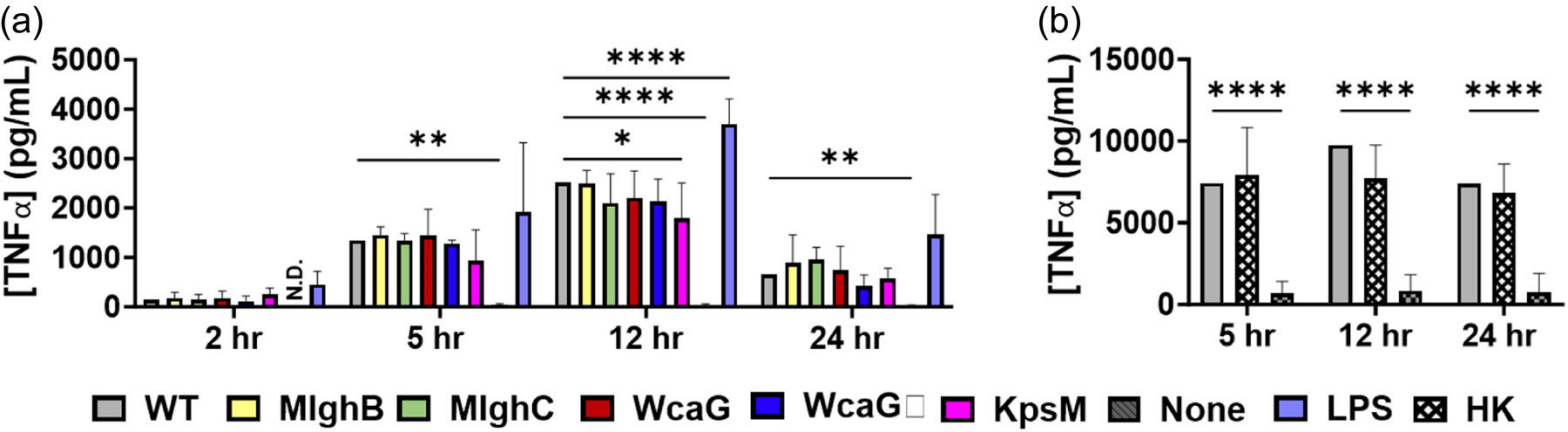
TNF⍺ induction in THP‐1 macrophages by  *Campylobacter jejuni* a‐capsular or capsular heptose mutants. Culture supernatants from THP‐1 cells cocultured with *C. jejuni* were assessed for the presence of TNF⍺ by ELISA. (a) Samples were taken at 2 h (*n* = 3), 5 h (*n* = 2), 12 h (*n* = 3), and 24 h (*n* = 4 for all except *mlghB* where *n* = 3) of coculture. (b) Samples were taken at 5 h (*n* = 3), 12 h (*n* = 3) and 24 h (*n* = 3) of coculture. Analysis was conducted by two‐way analysis of variance with subsequent Dunnett tests. Each biological replica is comprised of three technical replicates. Error bars represent standard deviation, all comparisons are to wild‐type. ND, none detected, *p < 0.05, **p < 0.001, ****p < 0.0001.

To further characterize the activation pattern of human THP‐1 cells in response to the capsule and its heptose, TNF⍺ secretion was assessed using ELISA over 24 h. Wild‐type *C. jejuni* led to elevated levels of TNF⍺ starting at 5 h, with a peak at 12 h (Figure 9a), with kinetics of activation mirroring that of LPS. TNF‐α induction was not dependent on bacterial viability (heat‐killed sample, Figure 9b) nor on the presence of heptose (heptose mutants, Figure 9a), and the capsule was only a minor contributor to the induction of TNF⍺ in these macrophages (*kpsM* mutant, Figure 9a).

**Figure 10:**
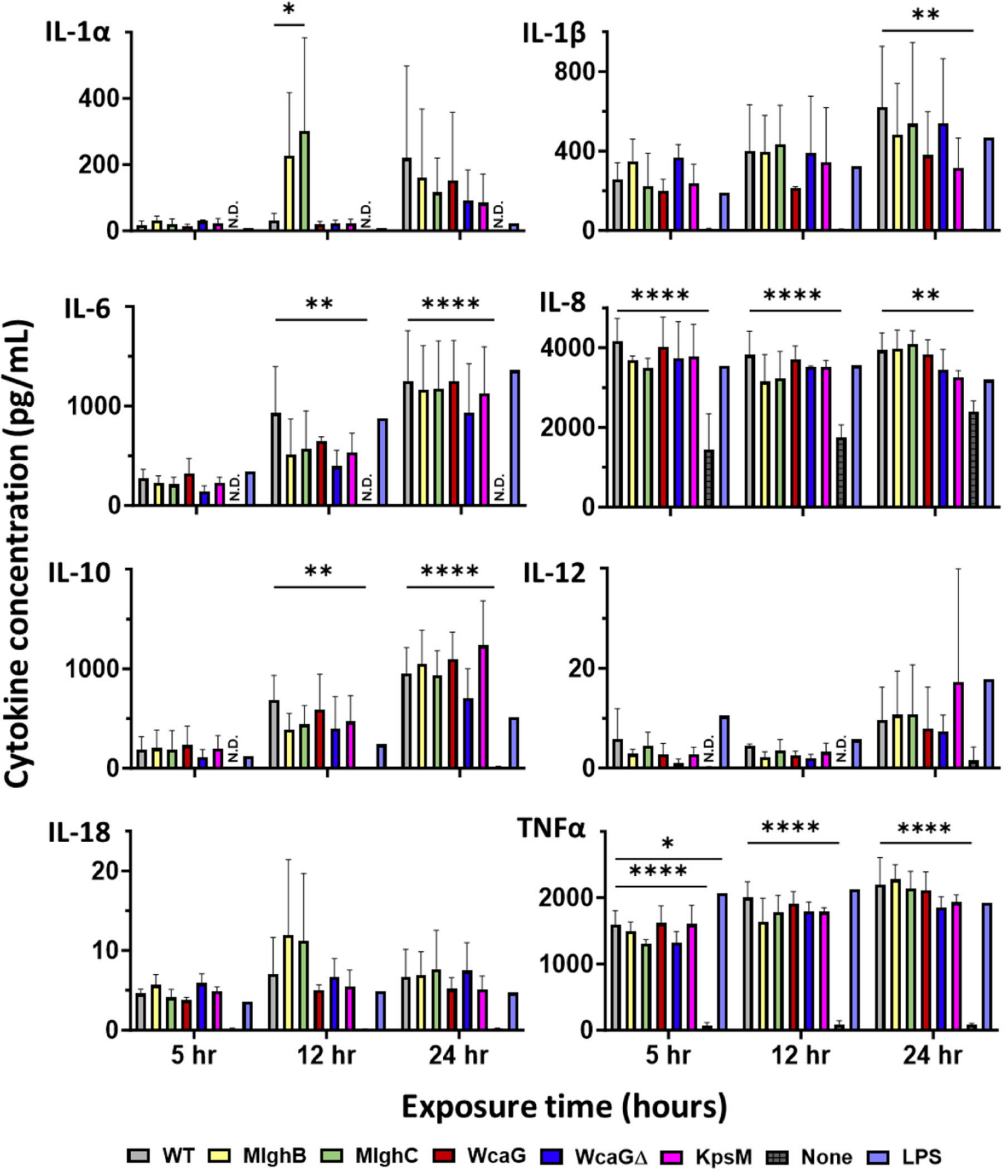
Cytokine output from human THP‐1 macrophage cocultured with  *Campylobacter jejuni*  capsular heptose mutants. Culture supernatants from THP‐1 cells cocultured with *C. jejuni* were assessed for the presence of cytokines using a multiplex assay. LPS was used as a positive control of activation. Cytokine names as indicated on each panel, except interleukin (IL)‐12 which stands for IL‐12p40 (*n* = 3) Biological replica for all times, with two technical determinations by multiplex for each. Statistical analysis was conducted by two‐way analysis of variance and subsequent Dunnett's test, error bars represent standard deviation. All comparisons are to wild‐type at each time point. ND, none detected, *p < 0.05, **p < 0.01,   ****p < 0.0001.

An 8‐cytokine multiplex conducted to delineate the full activation pattern of THP‐1 macrophages in response to *C. jejuni* showed that wild‐type *C. jejuni* caused induction of nearly all cytokines tested (Figure 10). Significant differences between wild‐type and the “macrophage only” samples were seen as early as after 5 h of coculture for TNF⍺ and IL‐8, followed by induction of IL‐6 and IL‐10 at 12 h. By 24 h, a significant increase in induction was seen for all tested cytokines, including IL‐1β. Generally, cytokine concentrations induced by wild‐type bacteria were similar to those observed with LPS, except for IL‐1⍺ and IL‐10 which were induced in higher amounts by *C. jejuni*.

Regarding the mutants, the TNF⍺ output from this multiplex matched the ELISA results, with the heptose mutants not differing significantly from the wild‐type (Figures 9, 10) and the *kspM* mutant displaying slightly diminished induction. For the other cytokines, the data indicate that the normal addition of the capsular heptose, or the whole capsule, had no effect, except for the *mlghC* mutant for IL‐1⍺ at 12 h. Overall, these data indicate that the capsule and its heptose do not significantly alter the induction of the 8 cytokines tested from THP‐1 human macrophages by *C. jejuni* NCTC 11168.

### 3.11 *C. jejuni* induces a strong but capsule‐independent pro‐inflammatory cytokine response in chicken macrophages

For lack of ELISA/multiplex reagents, the comparison of effector induction in chicken macrophages was conducted by qRT‐PCR after validation with human cells. Preliminary THP‐1 data showed upregulation of transcription for all cytokines tested (by a factor 2–30) in at least three of the four tested time points upon exposure to *C. jejuni* (Figure A5), with maximal transcript abundance at ~2.5 h for all cytokines. Also, the increase in transcript levels mirrored the increase in cytokine levels seen by multiplex, suggesting that the transcriptional assessment of the cytokine response is a valid surrogate to multiplex/ELISA and can be used for the chicken macrophages.

**Figure 11:**
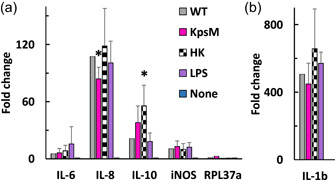
Quantitative real‐time polymerase chain reaction (qRT‐PCR) analysis of cytokine induction in chicken macrophages upon coculture with  *Campylobacter jejuni*. MQ‐NCSU cells were cocultured for 2 h 45 min with wild‐type *C. jejuni* used to live (WT) or heat‐killed (HK), the *kspM* mutant, LPS at 4 µg/mL, or in the absence of treatment (none). The equal viability of bacterial inoculum was determined by CFU counting (not shown). All data were analyzed as fold change compared with GAPDH and were normalized to the wild‐type average. (a) Interleukin (IL)‐6, IL‐8, IL‐10, iNOS, and RPL37a. (b) IL‐1β. MOI** =** 200, *n* **=** 3. Error bars represent standard deviation. No statistical significance was detected by two‐way analysis of variance relative to live wild‐type but statistical significance was reached by *T*‐test done within each cytokine data set with * for *p* **<** 0.05.

The chicken macrophage transcriptional data were examined relative to GAPDH and a second housekeeping gene (RPL37a) was also included (Maeß et al., 2010). There was no effect of any treatment/strain on RPL37a transcription, which confirms that GAPDH is an appropriate reference. Live wild‐type *C. jejuni* induced strong induction of all cytokines tested and of iNOS (used as positive control) (Figure 11a). The highest induction was for IL‐1β (505‐fold) followed by IL‐8 (107‐fold) and IL‐10 (21‐fold) (Figure 11a,b). The induction did not require bacterial viability and surprisingly, heat‐killed *C. jejuni* led to higher IL‐10 levels than live *C. jejuni* (*p* = 0.0497). While the capsule was not required for the induction of any cytokine or iNOS, the induction of IL‐8 was significantly lower in the *kpsM* mutant than in WT (*p* = 0.0289), with a decrease of 22%. Considering that the significance of IL‐8 for the a‐capsular mutant was low, it was deemed unlikely that individual capsule components would yield statistically significant differences, and the heptose mutants were thus not examined.

## 4 DISCUSSION

The behavior of wild‐type *C. jejuni* with regard to each phenotype tested, the role of the capsule and its heptose in eliciting or preventing each phenotype, and whether the contributions can be deemed deleterious or beneficial for *C. jejuni* survival, are summarized in Table 2.

**Table 2:** Summary of all phenotypes obtained in this study.

Phenotype	Features tested and strains used to assess their role				
	Live	Dead	Capsule	Heptose	Heptose and MeOPN
	Wild‐type	Wild‐type	*kpsM*	*mlghB mlghC*	*wcaG wcaGΔ*
**In vitro redox stress**					
Biofilm formation microaerobic	**++**	Na	**++**	**=**	**+**
Biofilm formation aerobic	**++++**	Na	**++++**	**++++**	**+++**
Nitrosative stress resistance	**+**	Na	**+++**	**+**	**++**
H_2_0_2_ resistance	**++**	Na	**+‐**	**+‐**	**+‐**
**Whole animal interaction**					
Fly gut colonization	**+++**	Na	**+++**	**+**	**++**
Virulence toward Galleria	**+++**	Na	‐‐	‐‐‐	‐‐‐
Chicken gut colonization*	**+++**	Na	**+**	**+++**	**+++**
**Adherence to macrophages**					
Chicken Mphg	**+**	Na	‐‐	**=**	‐ (MeOPN)
Human Mphg	**++**	Na	**=**	**=**	**=**
Human monocytes	**++**	Na	**=**	**+**	**=**
Murine Mphg*	**++**	Na	**‐**	**‐**	**+** (MeOPN)
**Uptake by macrophages**					
Chicken Mphg	**+**	Na	‐‐	**+++**	‐‐
Human Mphg	**++**	Na	‐‐‐	**=**	‐‐ (Heptose)
Human monocytes	**+**	Na	‐	**=**	‐‐ (MeOPN)
Murine Mphg*	**++**	Na	‐‐‐	**=**	**+** (MeOPN)
**Survival in macrophages**					
Chicken Mphg	**+**	Na	‐‐‐	‐	‐ (MeOPN)
Human Mphg	**++**	Na	‐	**++**	**+**
Human monocytes	**+**	Na	**=**	**++**	‐‐
Murine Mphg*	**++**	Na	‐	**=**	**=**
**Activation of macrophages**					
Chicken NO and iNOS production	**++++**	‐‐‐‐‐	**++**	‐‐	‐‐
Chicken ROS production	‐‐‐	**+**	**=**	**=**	**=**
Human ROS production	‐‐	**=**	**=**	**=**	**=**
Chicken cytokines production	**+++**	**++++**	**+ (**IL‐8 only)	Na	Na
Human cytokines production	**+++**	**+++**	**=**	**=**	**=**

*Note*: For live and dead  *Campylobacter jejuni* tests, the phenotype is relative to the “no exposure” control. For capsule and heptose tests, the phenotype is relatively to live wild‐type *C. jejuni*. The + and ‐ signs denote the role of the component tested in a phenotype, + and ‐ indicating the component supports an increase vs a decrease of that phenotype in the wild‐type context, respectively, and the varying numbers of signs reflect the intensity of the phenotype. Mphg, macrophage. *Data from our (Wong et al., 2015) study. Na, not applicable. Green boxes indicate the anticipated benefits of the capsule or heptose presence for *C. jejuni* survival. Pink boxes indicate anticipated deleterious effects for *C. jejuni* survival. Blank boxes indicate no effect or inability to conclude. The decisions for each box color integrate data from multiple phenotypes.

### 4.1 Capsule and biofilm mode of growth protect *C. jejuni* against aerobic insult

We showed that wild‐type *C. jejuni* NCTC 11168 significantly increased biofilm formation under aerobic conditions and this was consistent with other studies (Reuter et al., 2010; Wang et al., 2015). The increased biofilm formation likely protects *C. jejuni* from the oxygen, thus allowing prolonged survival. We also showed that the capsule and the heptose contribute to this process while MeOPN does not. Our findings are consistent with a recent report that the capsule is important for the aerobic protection of *C. jejuni* (Kim et al., 2023) and shows a new means whereby the capsule contributes to adaptation to oxygen stress by supporting growth as a biofilm.

### 4.2 The capsule and its modified heptose contribute to the survival of *C. jejuni* in the drosophila gastrointestinal track and limit virulence against *G. mellonella* larvae

The *D. melanogaster* and *G. mellonella* models are accepted to study host/bacteria interactions in the context of innate immunity because their innate immune system is reminiscent of the vertebrates', and they lack adaptive immunity (Browne et al., 2013; Hultmark, 2003; Igboin et al., 2012). However, by differing bacteria administration routes (ingestion vs injection), the fly model is commensal and the larvae model is pathogenic.

While the fly model is relevant to the environmental transmission of *C. jejuni*, to the best of our knowledge, it has not been used for *C. jejuni* to date. We showed that the capsule and the heptose were beneficial for *C. jejuni* NCTC 11168 to survive in the fly gut but were not essential and that the MeOPN was deleterious when present in a heptose‐less mutant. The *G. mellonella* model has been used for *C. jejuni* before, whereby the larvae succumb to infection by a wide variety of isolates (Emery et al., 2021; Gundogdu et al., 2011; Senior et al., 2011), as we also observed with our wild‐type strain. This implies that *C. jejuni* survives long enough in the larvae to exert its pathogenic effect despite its sensitivity to killing by serum and ROS. This suggests that the larvae's defenses including complement‐like proteins and a strong oxidative stress response are not sufficient to affect wild‐type *C. jejuni* (Gundogdu et al., 2011). The finding that all mutants devoid of capsule or heptose caused enhanced larvae death was unexpected considering their enhanced sensitivity to serum or H_2_O_2_ ((Wong et al., 2015) and this study). In a natural context, by limiting virulence, the capsule and heptoses may allow *C. jejuni* to preserve its host viability long enough to ensure transmission to a commensal host. It also suggests that pathogenicity in this model is imparted by virulence features other than the capsule and/or that sensitivity to serum or oxidative assaults may be circumnavigated by internalization in the plasmatocytes, akin to the ability of our mutants to survive in macrophages despite their oxidative burst.

The lack of statistical significance in the fly and larvae data likely reflects the relatively higher variability of the wild‐type data. We speculate that in situ variation of capsule composition may occur in a fraction of the population during the long time of the experiments. This could modulate *C. jejuni*'s survival and/or its pathogenic character, while such adjustments can't occur when the heptose genes are deleted.

### 4.3 Differential effects of the capsule on adherence, uptake, and intracellular survival allow *C. jejuni* NCTC 11168 to survive better in humans than in chicken macrophages

While the insect models above encompass interactions with humoral and cellular innate immune defenses, the role of the capsule and its heptose in the direct interactions of *C. jejuni* with innate immune cells remained to be assessed, including the fate of *C. jejuni* upon interaction with macrophages and the effect that *C. jejuni* had on the macrophages.

In contrast to the capsule of many pathogens (Gong et al., 2022; Ni et al., 2020; Wen et al., 2016), the *C. jejuni* capsule only offers limited protection against phagocytosis by macrophages (Kim et al., 2018; Myszewski & Stern, 1991; Sikic Pogacar et al., 2009; Wassenaar et al., 1997), including in our study with chicken and human macrophages where we only observed a slight capsule‐mediated decrease in adherence (chicken cells only) or in uptake (chicken and human cells). Furthermore, consistent with many studies that reported killing within ~6 h of *C. jejuni* after uptake by macrophages (Myszewski & Stern, 1991; Šikić Pogačar et al., 2009; Wassenaar et al., 1997; Wong et al., 2015), we recovered live *C. jejuni* only up to 3 h after phagocytosis. The capsule appeared detrimental to intracellular survival, especially in chicken macrophages. Indeed, while high and equivalent levels (~10E5 CFU/mL) of the a‐capsular mutant were recovered in both species (Figure 5d,f), ~1.8 log less wild‐type bacteria were recovered in chicken macrophages vs only a ~0.4 log decrease in human macrophages. This suggests that encapsulated internalized *C. jejuni* would be eliminated better by chicken than by human macrophages

Overall, accounting for all differences highlighted at each step of the interactions with each macrophage species (summarized in Table 2), the encapsulated wild‐type *C. jejuni* may survive temporarily in human macrophages, which would protect it from extracellular defenses and may enhance its pathogenicity in human hosts, while the capsule limits this possibility in chicken macrophages. It remains, however, that the capsule is detrimental to the survival of wild‐type *C. jejuni* NCTC 11168 within macrophages in both species and thus, intervention strategies that would eliminate the capsule may impair clearance by macrophages. This underscores the importance of exploring our encapsulated mutants for potential intervention.

### 4.4 The activities of *mlghB* and *mlghC* mitigate the deleterious effect of the capsule on intracellular survival in human macrophages while they support faster killing by chicken macrophages

The wild‐type capsule composition seems optimized to permit host colonization, especially in a commensal context, as mutations of the heptose synthesis pathway drastically reduced colonization of the chicken gut, much more so than the outright capsule removal (Wong et al., 2015). We surmise that it is also optimal to mitigate the slightly detrimental effect of the capsule during interactions with macrophages and may affect the interactions in a species‐specific manner. Indeed, the normal activities of *mlghB* and *mlghC* enhance intracellular survival of wild‐type *C. jejuni* in human macrophages and monocytes but not in chicken cells (Table 2). Consistently, levels of *C. jejuni* associated with chicken MQ‐NCSU macrophages are very low in vivo (Taha‐Abdelaziz et al., 2019). This suggests that the *mlghB/C* activity may support interactions with other types of cells to explain their favorable role in chicken gut colonization, and accordingly, the active *mlghB/C* genes are required for invasion and survival within human epithelial cells (Wong et al., 2015). The *wcaG* and *wcaGΔ* mutants also showed species‐specific effects on macrophages although not as strong as the *mlghB/C* mutants and did not affect human epithelial cell invasion (Wong et al., 2015).

### 4.5 *C. jejuni* causes drastically different oxidative and nitrosative stress responses in macrophages, with capsule‐induced nitrosative stress partly mitigated by its heptose

The chicken macrophages produced very high levels of NO in response to *C. jejuni* and consistently, showed strong induction of the iNOS gene that is responsible for NO production. Similar findings have also been reported in murine bone marrow‐derived macrophages (Iovine et al., 2008). The NO burst was dependent on bacterial viability, which was somewhat incongruent with the fact that purified *C. jejuni* LOS and outer membrane proteins can induce NO in MQ‐NCSU chicken macrophages (Taha‐Abdelaziz et al., 2020). Though these components may be exposed during infection in vivo if/when capsule expression is downregulated (Guerry et al., 2012), the capsule is produced under our in vitro conditions (Wong et al., 2015) and may mask these components. Surprisingly, *C. jejuni* viability was not required for induction of iNOS, suggesting that additional inputs relying on live *C. jejuni* are needed for NO production. Indeed, iNOS utilizes nicotinamide‐adenine‐dinucleotide phosphate (NADPH) which is also used by NADPH oxidase (NOX) to make superoxide and by redox enzymes that protect against ROS (Pizzorno, 2014). Thus, the competition for NADPH under oxidative stress limits its availability for iNOS and limits NO production despite iNOS induction when strong ROS production is induced (as seen with heat‐killed bacteria). When living *C. jejuni* tones down ROS production by macrophages, NADPH is not limiting, thus allowing iNOS activity and NO production.

As suggested by the in vitro nitrosative stress data, the 0.4 mM NO released by chicken macrophages is bound to take their toll on *C. jejuni*. Our data highlight two strategies that converge in limiting and in resisting the macrophage nitrosative attack: the capsule‐ and heptose‐mediated limitation in sensitivity to nitrosative stress and the heptose‐mediated mitigation of the capsule‐induced nitrite production. These protections do not exclude the role of proteins that *C. jejuni* expresses in combating the deleterious effects of NO (Atack & Kelly, 2008; Elvers et al., 2004; Pittman et al., 2007).

As for ROS, no activation of ROS release was observed, but dampening of ROS production occurred in all macrophages. This required bacterial viability but was independent of capsule or heptose. The dampening seemed more efficient in human cells (~2/3 of the basal ROS levels) than in chicken macrophages (~half only). Mirroring these data, other *C. jejuni* strains also moderated ROS induction in human intestinal epithelial cells (Hong et al., 2023) and the reduction was linked to decreased transcription of Nox1. Our H_2_O_2_ exposure data showed that *C. jejuni* is not overly sensitive to short‐term and limited oxidative stress and that the capsule and the heptose only provide limited protection. Protection is likely afforded by reductases (Atack & Kelly, 2008) but is overall weak once a high oxidant concentration is reached. It is thus consistent to observe that *C. jejuni* dampens the ROS response to begin with.

Overall, we highlighted a capsule‐ and heptose‐independent mechanism to prevent/minimize the oxidative assault, associated with minimal protection by the capsule and heptose when the assault occurs. This is different from the capsule‐mediated nitrosative burst reported above, where heptose contributed to resistance to that assault. This suggests that the nitrosative and oxidative stress responses involve different mechanisms of regulation. This is also different from the oxygen‐induced biofilm growth that the capsule and heptose contribute to. In this context, the capsule and heptose may provide protection mostly by creating the extracellular matrix needed to stabilize the biofilms, rather than by direct contribution to resistance to oxidation as seen in the H_2_O_2_ exposure assay.

### 4.6 Wild‐type *C. jejuni* NCTC 11168 triggers a strong cytokine response from both human and chicken macrophages, but the capsule and its heptose are not required for the process

Our multiplex and ELISA analyses show strong activation of human macrophages in response to wild‐type *C. jejuni* NCTC 11168. The cytokines were released with differential kinetics, starting with TNF⍺ and IL‐8, followed by IL‐6 and IL‐10 at 12 h and IL‐1β at 24 h. These data agree with studies on other *C. jejuni* strains (Bouwman et al., 2014; Jones et al., 2003; Siegesmund et al., 2004). The strong induction of anti‐inflammatory IL‐10 suggests a counterbalancing act to prevent an overt reaction that could eliminate *C. jejuni*. Also, though IL‐6 is generally accepted as a pro‐inflammatory cytokine, it has context‐dependent anti‐inflammatory activity (Fuster & Walsh, 2014), cooperating with IL‐10 to control the level of pro‐inflammatory cytokines (Xing et al., 1998) and dampening macrophages activation to protect surrounding tissues against the deleterious effects of inflammation (Luig et al., 2015). This is consistent with the moderate levels of pro‐inflammatory IL‐1β observed after IL‐10 and IL‐6 are induced, as well as the lack of induction of IL‐12p40, IL‐18, and IL‐1α. This concerted induction of IL‐10 and IL‐6 may allow *C. jejuni* to persist, although for a limited time, in human macrophages and thus in the host, by preventing a broad systemic inflammatory reaction. However, the capsule and its heptose played no role in cytokine induction from human macrophages.

As for chicken macrophage activation, the strong induction of the iNOS positive control correlated well with the strong NO burst seen with wild‐type live *C. jejuni*. We observed very strong induction (5–505‐fold) of IL‐6, IL‐8, IL‐10, and IL‐1ß, the highest induction being for IL‐8 and IL‐1ß. Our findings agree with prior studies performed on chicken MQ‐NCSU macrophages with *C. jejuni* strain 81116 or with campylobacter‐derived ligands (Taha‐Abdelaziz et al., 2020; Vaezirad et al., 2017).

The capsule only contributed to enhancing IL‐8 induction in chicken macrophages but was not essential. While activation of pro‐inflammatory cytokines in a commensal context was surprising, it may be that the concomitant activation of IL‐6 and IL‐10 helps maintain the inflammatory response at low levels in vivo, though the IL‐6 induction was the lowest of all cytokines tested (five‐fold). The fact that strong cytokine induction did not require bacterial viability nor the capsule suggests activation by stable surface‐bound pathogen‐associated molecular patterns that are not masked by the capsule. This may include the flagella that can signal through Toll‐like receptor TLR5 and induce iNOS in MQ‐NCSU macrophages (Vaezirad et al., 2017).

### 4.7 Species‐and strain‐specific capsule‐based interactions with macrophages in other studies

Compared with our human and chicken data reported here, our prior studies with murine RAW 264.7 macrophages show yet again different roles of the capsule and its heptose on *C. jejuni*'s fate during the interactions (Wong et al., 2015, and Table 2). This calls for caution when generalizing murine‐based data to human pathogenesis or chicken‐based intervention strategies with regard to *C. jejuni*.

Comparative studies of a‐capsular *C. jejuni* mutants during interactions with immune cells from different species are very scarce and none exists for heptose‐less mutants. For example, the capsule of *C. jejuni* 81‐176 has been widely studied for its role in virulence in ferret models and was shown to be immunoprotective in human THP‐1 macrophages (Bacon et al., 2001; Stephenson et al., 2013) but the role of heptose is unknown. This immunosuppression is not occurring in our study with strain NCTC 11168, though its capsule can mitigate cytokine production in dendritic cells (Rose et al., 2012). Also, a recent study showed that the *C. jejuni* NCTC 11168H capsule diminished survival slightly in primary human macrophages but not in chicken macrophages (Kim et al., 2018) and that the lack of MeOPN decreased phagocytosis but did not impact survival in either macrophage. This differs from our data and differences between the two studies could be because we only tested MeOPN in heptose‐less mutants, and the wild‐type capsule of NCTC 11168H may contain MeOPN, while the wild‐type NCTC 11168 does not (Wong et al., 2015). Also, hyperflagellation occurs in the NCTC 11168H strain and the flagella affect signaling in MQ‐NCSU macrophages (Vaezirad et al., 2017), which can affect *C. jejuni*'s fate and mask the effect of other virulence factors. Nevertheless, concordance was observed regarding the lack of requirement of capsule and MeOPN for cytokine induction in chicken and human macrophages, and an interesting murine‐specific quenching of IL‐6 and IL‐10 transcription mediated by the capsule and MeOPN was highlighted.

Overall, the paucity of information and the differences in outcomes observed with various *C. jejuni* strains and types of macrophages studied underscore the need for concurrent comparative studies in relevant host cells, especially in human and chicken cells where potential intervention against *C. jejuni* would be applicable and to which murine data may not extrapolate.

## 5 CONCLUSIONS

Our data highlight the multifaceted processes involved during the interaction of *C. jejuni* with various hosts and host components that result in vast differences in behavior across different models, species, and Campylobacter strains. Importantly, in several of our assays, the effect of the heptose and its modification pathway was different from that of the whole capsule, thus providing novel functional information not only on the capsule but also on the heptose (Table 2)**.**

Specifically, we showed that the capsule and heptose are important to mitigate exposure and sensitivity to redox stresses and contribute to survival in the intestinal tract of *D. melanogaster*, akin to our observations that the capsule, and even more prominently the heptose, were key factors for chicken gut colonization (Table 2). We also showed that while *C. jejuni* NCTC 11168 is pathogenic in the *G. mellonella* model, the capsule and heptose unexpectedly slightly toned down the virulence.

As for interactions with human and chicken macrophages, highly similar behaviors were observed despite the pathogenic vs commensal relationship in whole hosts, including the limited anti‐phagocytic capacity of the capsule and its slightly detrimental effect on intracellular survival, the strong induction of both pro‐ and anti‐inflammatory cytokines and the active dampening of the ROS response that relied on bacterial viability but was capsule‐ and heptose‐independent. Species‐specificity was mostly observed when dissecting the fate of *C. jejuni* at different steps of the interactions, with differential roles of the various genes of the heptose modification pathway.

Overall, the capsule and its heptose are double‐edged swords that can protect *C. jejuni* against some host defenses while enhancing the activation of others. As highlighted in Table 2, the capsule has globally more beneficial than detrimental effects for *C. jejuni*, and the heptose and its modification pathway can mitigate some of the capsule's detrimental effects. This suggests that capsule production and heptose incorporation may be modulated at different stages of the infection.

## AUTHOR CONTRIBUTIONS

**Matthew Myles**: Methodology; investigation; formal analysis; validation; writing—original draft; writing—review and editing; visualization; data curation; supervision. **Heba Barnawi**: Validation; formal analysis; writing—original draft; writing—review and editing; methodology; visualization; data curation. **Mahmoud Mahmoudpour**: Investigation; writing—review and editing; methodology. **Sargon Shlimon**: Investigation; writing—review and editing; methodology. **Adrienne Chang**: Investigation; methodology; writing—review and editing. **Daniel Zimmermann**: Investigation; writing—review and editing; formal analysis. **Chiwon Choi**: Methodology; investigation; writing—review and editing. **Najwa Zebian**: Methodology; supervision; writing—review and editing. **Carole Creuzenet**: Conceptualization; methodology; data curation; supervision; validation; visualization; resources; writing—review and editing; writing—original draft; project administration; funding acquisition; formal analysis; investigation.

## CONFLICT OF INTERESTS STATEMENT

None declared.

## ETHICS STATEMENT

None required.

## Figures and Tables

**Figure A3:**
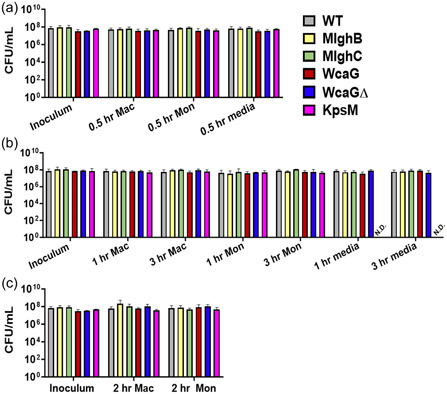
Viability of the inoculum used for interaction studies between  *Campylobacter jejuni* and human macrophages and monocytes. (a) For adherence studies. (b) For uptake studies. (c) For intracellular studies. In each case, the inoculum was enumerated by CFU counting at the time of coculture setup, and at the time of removal of the nonadhered or external bacteria, as applicable. Mac: macrophages. Mon: monocytes. The adherence, uptake, and intracellular data are presented in Figure 6.

**Figure A4:**
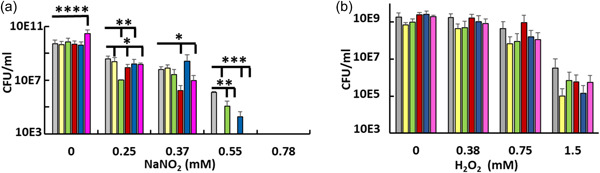
Nonnormalized data for nitrosative (a) and oxidative (b) exposure assays. The normalized data are presented in Figures 7b and 8c. When no bacteria were recovered, the value was assigned to the detection limit of this assay (10E3 CFU/mL). Data gathered from six biological replicates comprising various strain combinations, resulting in *n* = 3–12 depending on the strains and the concentrations tested. No statistical significance obtained by two‐way analysis of variance on either assay. Statistical significance obtained by *T*‐tests in NaNO_2_ exposure assay only. For both panels, the error bars represent the standard deviation and all comparisons are to wild‐type with *p < 0.05; **p < 0.01; ***p < 0.001, ****p < 0.0001.

## Data Availability

All data generated or analyzed during this study are included in this published article and its Appendix A.

## Appendix

Figures A1, A2, A3, A4, A5
